# Suppression of CCL2 angiocrine function by adrenomedullin promotes tumor growth

**DOI:** 10.1084/jem.20211628

**Published:** 2022-11-14

**Authors:** Akiko Nakayama, Kenneth Anthony Roquid, András Iring, Boris Strilic, Stefan Günther, Min Chen, Lee S. Weinstein, Stefan Offermanns

**Affiliations:** 1 Department of Pharmacology, Max Planck Institute for Heart and Lung Research, Bad Nauheim, Germany; 2 Bioinformatics and Deep Sequencing Platform, Max Planck Institute for Heart and Lung Research, Bad Nauheim, Germany; 3 Metabolic Disease Branch, National Institute of Diabetes and Digestive and Kidney Diseases, National Institutes of Health, Bethesda, MA; 4 Center for Molecular Medicine, Goethe University Frankfurt, Frankfurt, Germany; 5 Cardiopulmonary Institute, Bad Nauheim, Germany; 6 German Center for Cardiovascular Research, Bad Nauheim, Germany

## Abstract

Within the tumor microenvironment, tumor cells and endothelial cells regulate each other. While tumor cells induce angiogenic responses in endothelial cells, endothelial cells release angiocrine factors, which act on tumor cells and other stromal cells. We report that tumor cell–derived adrenomedullin has a pro-angiogenic as well as a direct tumor-promoting effect, and that endothelium-derived CC chemokine ligand 2 (CCL2) suppresses adrenomedullin-induced tumor cell proliferation. Loss of the endothelial adrenomedullin receptor CALCRL or of the G-protein G_s_ reduced endothelial proliferation. Surprisingly, tumor cell proliferation was also reduced after endothelial deletion of CALCRL or G_s_. We identified CCL2 as a critical angiocrine factor whose formation is inhibited by adrenomedullin. Furthermore, CCL2 inhibited adrenomedullin formation in tumor cells through its receptor CCR2. Consistently, loss of endothelial CCL2 or tumor cell CCR2 normalized the reduced tumor growth seen in mice lacking endothelial CALCRL or G_s_. Our findings show tumor-promoting roles of adrenomedullin and identify CCL2 as an angiocrine factor controlling adrenomedullin formation by tumor cells.

## Teaser

The authors identify a tumor-suppressing angiocrine function of CCL2 which is inhibited by tumor-derived adrenomedullin acting via its receptor on endothelial cells. Disinhibition of endothelial CCL2 results in reduced tumor progression through inhibition of adrenomedullin formation by tumor cells.

## Graphical Abstract

**Figure:**
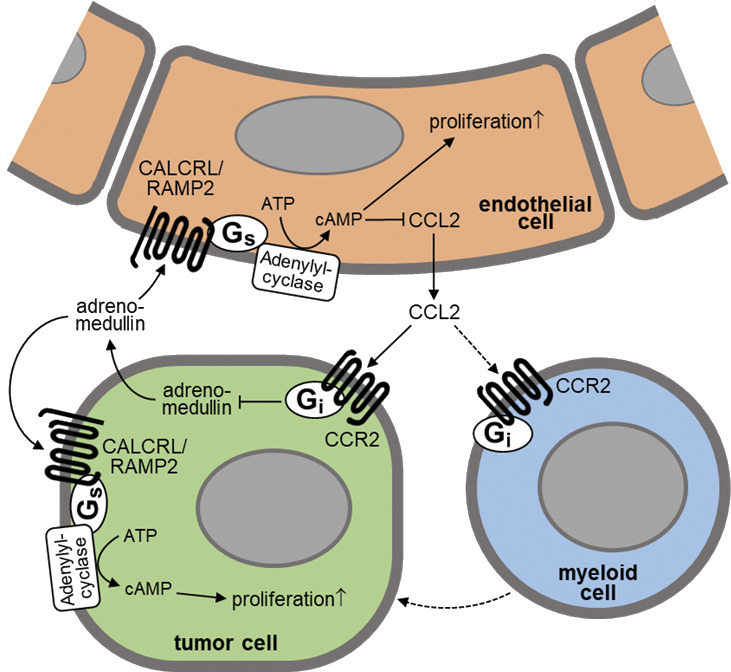

## Introduction

Tumor cells and endothelial cells interact in multiple ways within the tumor microenvironment, which critically contributes to tumor progression, metastatic dissemination, and immune surveillance of cancer cells, as well as to the response to therapies (De Palma et al., 2017; Maman and Witz, 2018; Nagl et al., 2020). It is well established that both tumor cells and the tumor microenvironment regulate endothelial cells through various pro- and anti-angiogenic factors. However, within the tumor stroma, endothelial cells not only respond to tumor-derived angiogenic factors but have also been recognized during the last decade to produce and release factors that regulate neighboring cells including tumor cells. These angiocrine factors include growth factors, interleukins, or chemokines, and have been shown to critically contribute to cancer progression and metastasis (Butler et al., 2010; Maishi et al., 2019).

Adrenomedullin is a regulatory peptide that was first isolated from human adrenal tumor (Kitamura et al., 1993). Multiple tumors have been shown to express adrenomedullin, and it is believed to function as a potent autocrine or paracrine factor that promotes tumor cell survival and lymphangiogenesis (Klein and Caron, 2015; Larrayoz et al., 2014; Zudaire et al., 2003). Adrenomedullin has also been proposed as a mediator of tumor angiogenesis (Nikitenko et al., 2006). This is based on the ability of adrenomedullin to induce pro-angiogenic effects in endothelial cells in vitro (Kim et al., 2003; Martinez et al., 2002; Zhang et al., 2017) as well as on the observation that the expression levels of adrenomedullin in tumors correlate with tumor angiogenesis in vivo (Hague et al., 2000; Martinez et al., 2002; Oehler et al., 2002). However, evidence for a direct pro-angiogenic effect of adrenomedullin in tumors is missing and how adrenomedullin activity is regulated in tumors is poorly understood.

Adrenomedullin exerts its cellular effects by activating the G-protein–coupled receptor calcitonin receptor–like receptor (CALCRL), which requires the presence of receptor activity–modifying proteins 2 or 3 (RAMP2 or RAMP3) to function as an adrenomedullin receptor (Poyner et al., 2002; Schonauer et al., 2017; Vazquez et al., 2020). The adrenomedullin receptor is coupled to the G-protein G_s_ which activates adenylyl cyclase resulting in increased formation of cAMP, a pathway which has been shown to increase endothelial barrier function and nitric oxide formation and to mediate anti-inflammatory effects in endothelial cells (Iring et al., 2019; Moy et al., 1998; Nakayama et al., 2020).

Based on co-culture experiments in vitro as well as on in vivo experiments, we show that tumor cell–derived adrenomedullin exerts a pro-angiogenic effect on endothelial cells, which is mediated by the G_s_-coupled adrenomedullin receptor. In addition, we show that adrenomedullin acting on endothelial cells further stimulates formation of adrenomedullin in tumor cells by inhibiting the endothelial formation and release of CCL2, which acts as an angiocrine factor to inhibit adrenomedullin expression by tumor cells. Increasing endothelial CCL2 formation may therefore be a strategy to decrease tumor growth.

## Results

### Loss of endothelial G_s_-mediated signaling reduces tumor progression

**Figure S1.**
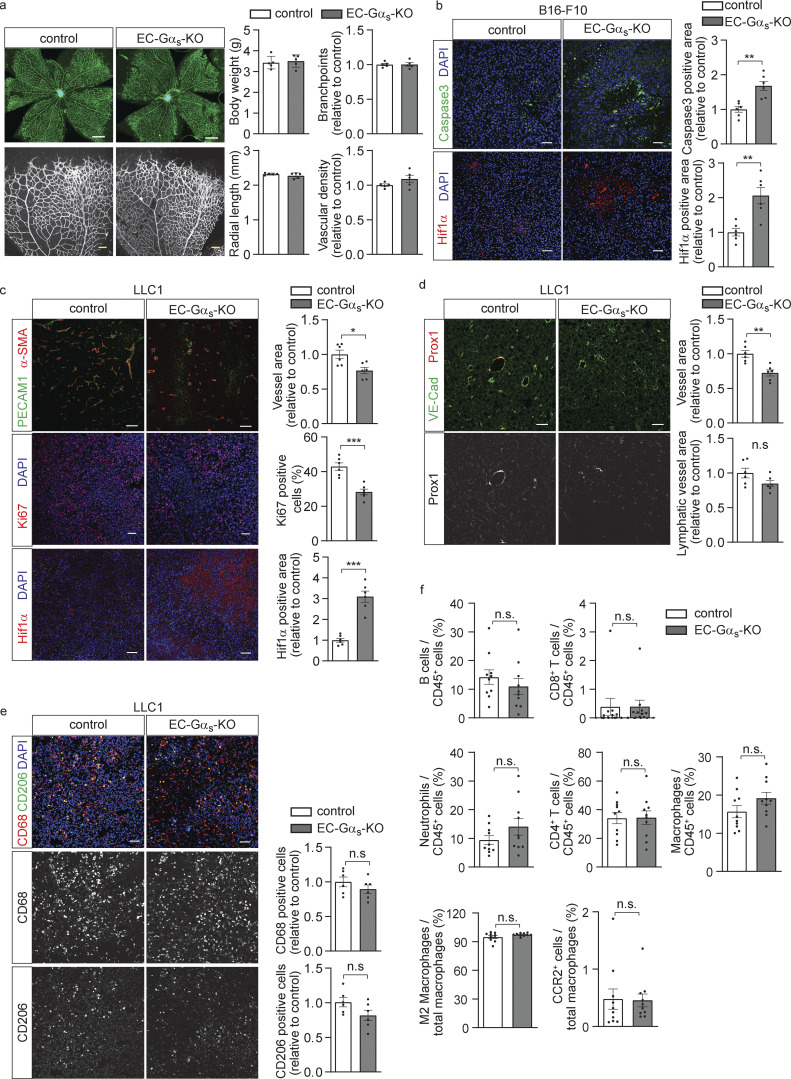
**Effects of endothelial loss of Gα**_**s**_ **on retinal angiogenesis and tumor angiogenesis. (a)** Whole mount retinae of control and EC-Gα_s_-KO mice at P7 stained with Isolectin B4. Quantification of vasculature parameters are presented as bar diagrams (*n* = 5 mice). **(b)** Immunohistochemistry of B16-F10 tumors dissected from control or EC-Gα_s_-KO mice. Sections were analyzed for a marker of apoptosis (cleaved caspase3; green) or of hypoxia (Hif1α; red) and were stained with DAPI (blue; *n* = 6 mice for each genotype). **(c–e)** Immunohistochemistry of LLC1 tumors dissected from control or EC-Gα_s_-KO mice. Sections were analyzed for markers of endothelial cells (PECAM1 or VE-Cad; green), lymphatic endothelial cell (Prox1; red), perivascular cells (α-SMA; red), proliferating cells (Ki67; red), hypoxia (Hif1 α; red), as well as M1 and M2 macrophages (CD68 and CD206), and were stained with DAPI (blue). The bar diagrams show the statistical evaluation (*n* = 6 mice for each genotype). **(f)** Flow cytometric analysis of immune cells in tumors. LLC1 tumors dissected from control or EC-Gα_s_-KO mice were dissociated, and populations of neutrophils, B cells, CD8^+^ T cells, CD4^+^ T cells, and macrophages were analyzed by determining the expression of Ly6G, CD8, CD4, and F4/80, respectively. F4/80 expressing macrophage were also evaluated for their expression of the M2 marker CD206 or of the CCL2 receptor CCR2 (*n* = 10 mice per group). Bar length: 500 μm, 100 μm (a); 50 μm (b–e). Data represent mean values ± SEM; *, P ≤ 0.05; **, P ≤ 0.01; ***, P ≤ 0.001 (two-tailed Student’s *t* test).

**Figure 1.**
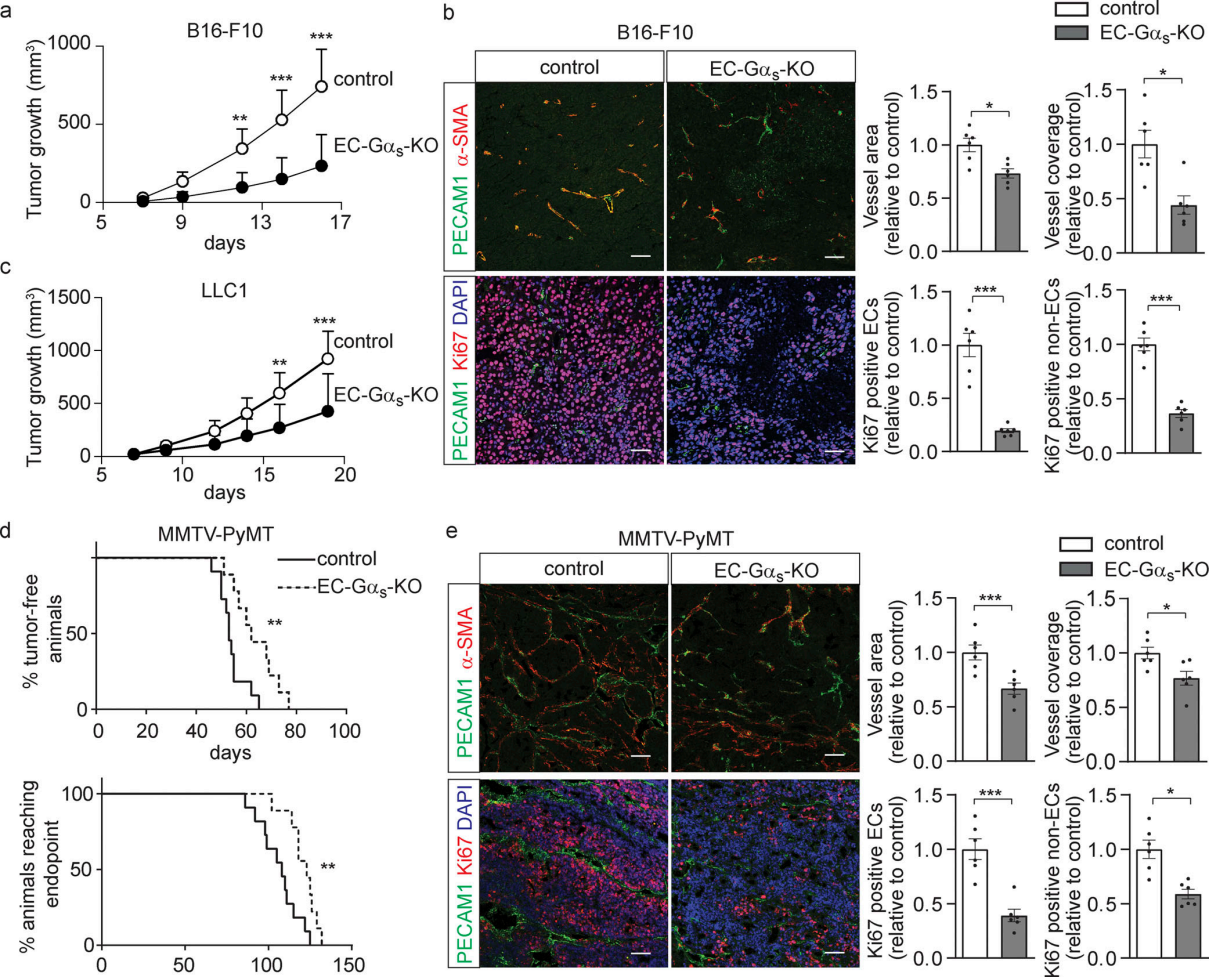
**Loss of endothelial Gα**_**s**_ **reduced primary tumor growth. (a–c)** B16-F10 (a and b) or LLC1 (c) tumor cells were injected subcutaneously in control and EC-Gα_s_-KO mice, and tumor growth was determined (*n* = 9 mice; a and c). B16-F10 tumor sections were analyzed for markers of endothelial cells (PECAM1; green), perivascular cells (α-SMA; red) or for proliferating cells (Ki67; red), and were stained with DAPI (blue). The bar diagrams (b) show the statistical evaluation (*n* = 6 mice per group). **(d and e)** Control or EC-Gα_s_-KO mice crossed with MMTV-PyMT mice were monitored for occurrence of tumors as well as for the time point at which they had reached the endpoint of the experiment (d), and tumor sections were analyzed by immunohistochemistry (e) using antibodies recognizing endothelial cells (PECAM1; green), perivascular cells (α-SMA; red) and proliferating cells (Ki67; red), and were stained with DAPI (blue). The bar diagrams (e) show the statistical evaluation (*n* = 6 mice for each genotype). Bar length (b and e): 50 μm. Data represent mean values ± SEM; *, P ≤ 0.05; **, P ≤ 0.01; ***, P ≤ 0.001 (two-way ANOVA and Bonferroni’s post hoc test [a and c], two-tailed Student’s *t* test [b and e], and Gehan-Breslow-Wilcoxon test [d]).

To investigate the role of endothelial G_s_-mediated signaling in vivo, we analyzed mice with tamoxifen-inducible endothelium-specific deletion of Gα_s_, the α-subunit of the G-protein G_s_ (Tek-CreERT2;*Gnas*^*flox*/flox^, herein referred to as EC-Gα_s_-KO mice; Iring et al., 2019). Loss of endothelial Gα_s_ had no effect on the development of the retinal vasculature at postnatal day 7 (P7; Fig. S1 a). However, when we examined endothelial Gα_s_ function in syngeneic tumor models, we found that growth of B16-F10 melanoma was significantly decreased in EC-Gα_s_-KO mice compared to control animals (Fig. 1 a). Tumors in EC-Gα_s_-KO mice showed reduced proliferation of endothelial and non-endothelial cells, reduced vessel density, reduced vessel coverage, increased cell death, and an increased hypoxic area (Fig. 1 b and Fig. S1 b). Lewis lung carcinoma (LLC1), when grown in EC-Gα_s_-KO mice, also showed reduced growth as well as reduced cell proliferation, reduced vessel density without affecting lymphatic vessel area, and an increased hypoxic area (Fig. 1 c and Fig. S1, c and d), whereas immune cell infiltration was comparable between control and EC-Gα_s_-KO mice (Fig. S1, e and f). We then studied a spontaneous breast cancer model after crossing control or EC-Gα_s_-KO mice with a mouse line in which the polyoma middle T-antigen expression is driven by the mouse mammary tumor virus promoter (MMTV-PyMT). Endothelial loss of Gα_s_ delayed tumor growth in MMTV-PyMT mice (Fig. 1 d), and this effect was accompanied by reduced vessel density and reduced proliferation of endothelial and non-endothelial cells (Fig. 1 e). These data indicate that endothelial Gα_s_ plays a critical role in tumor growth.

### The adrenomedullin receptor CALCRL as well as G_s_ are required for endothelial and tumor cell proliferation

**Figure 2.**
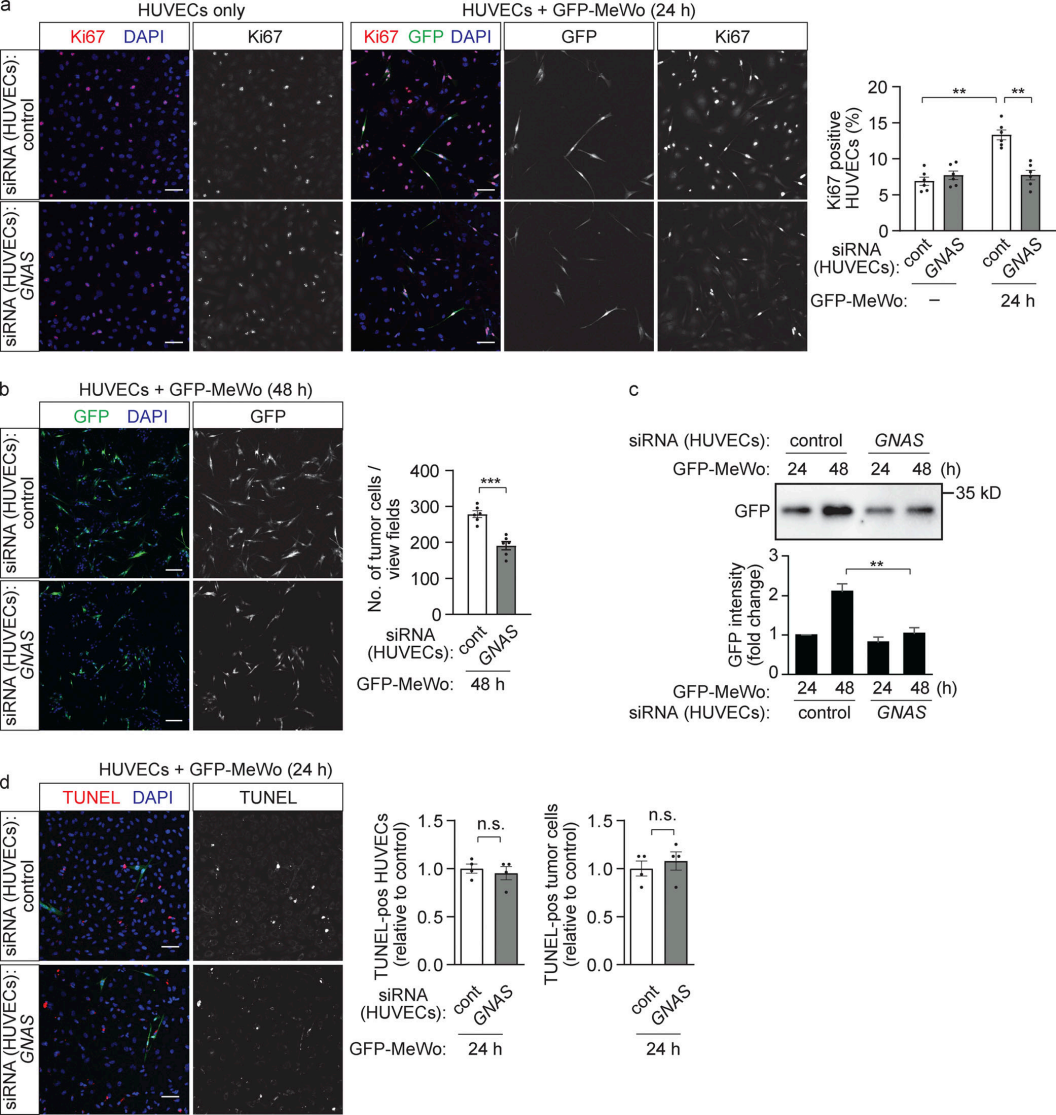
**Loss of endothelial Gα**_**s**_ **reduced endothelial and tumor cell growth in vitro. (a–d)** HUVECs were transfected with control siRNA or siRNA directed against *GNAS*, the gene encoding Gα_s_, and were cultured alone or together with GFP-expressing human MeWo melanoma cells (GFP-MeWo) for the indicated time periods. Proliferating cells were stained with anti-Ki67 antibody (a; red), or apoptotic cells were detected using the TUNEL assay (d; red). Cells were counterstained with DAPI (a, b, and d; blue). The number of Ki67-positive HUVECs (a), GFP-expressing tumor cells (b), or TUNEL-positive cells (d) was determined by immunofluorescence, or the expression level of GFP was determined by Western blot analysis as an indicator of tumor cell growth (c). The bar diagrams show the statistical evaluation (a and b, *n* = 3 independent experiments; d, *n* = 2 independent experiments) or represent the relative densitometric values based on Fiji software (c; *n* = 3 independent experiments). Bar length: 100 µm (a, b, and d). Data represent mean values ± SEM; **, P ≤ 0.01; ***, P ≤ 0.001 (two-way ANOVA and Bonferroni’s post hoc test [a and c] and two-tailed Student’s *t* test [b and d]).

**Figure S2.**
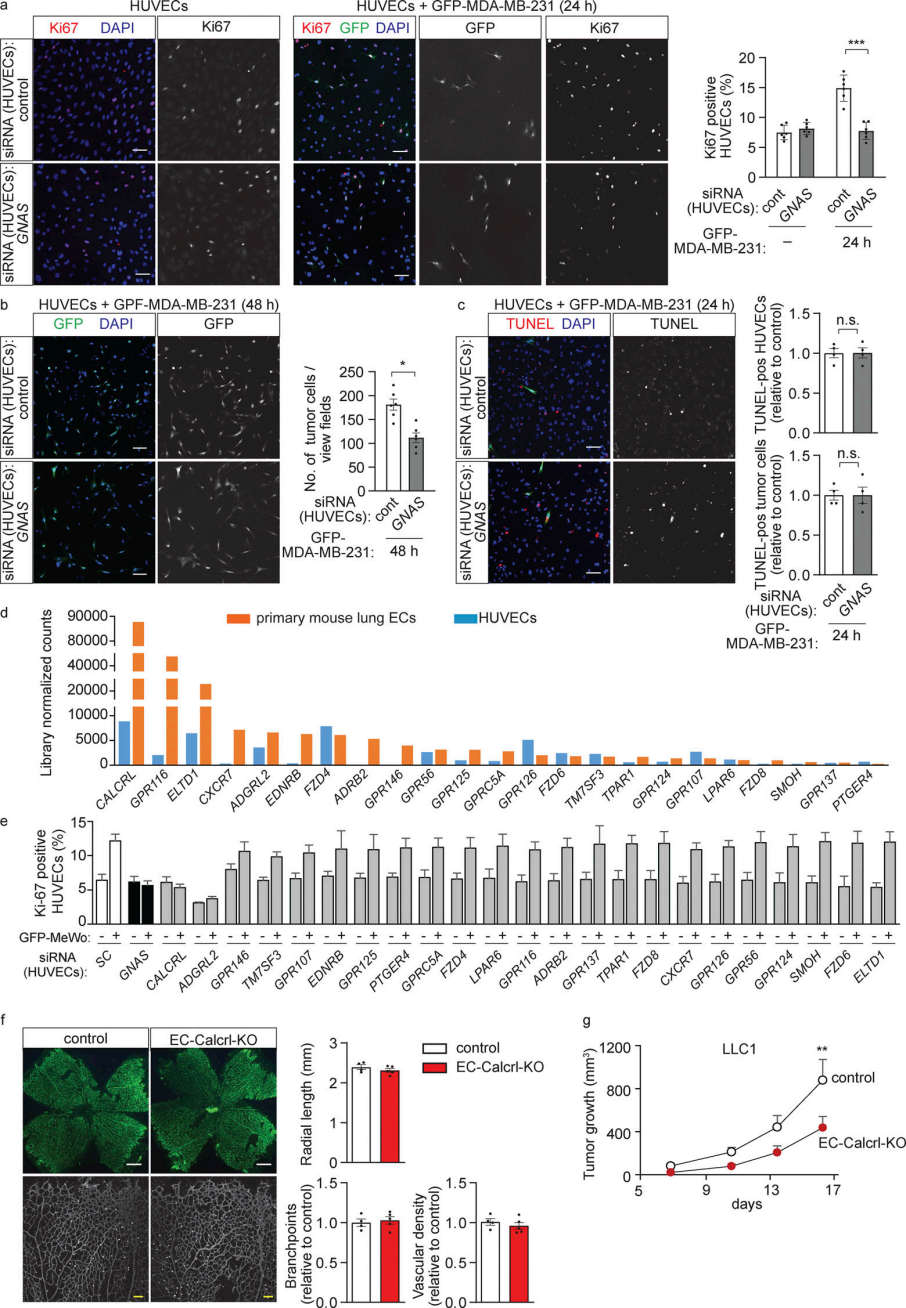
**Role of endothelial Gα**_**s**_ **or CALCRL in vitro and in vivo. (a–c)** HUVECs were transfected with control siRNA or siRNA directed against *GNAS* and were cultured alone (a, left) or with GFP-expressing human MDA-MB-231 breast cancer cells (GFP-MDA-MB-231) for 24 h (a [right] and c) or for 48 h (b). Endothelial cell proliferation was determined by staining for Ki67 (a; *n* = 3 independent experiments), the number of GFP-expressing tumor cells was determined by immunofluorescence (b; *n* = 3 independent experiments) or apoptotic cells were detected using the TUNEL assay (c; *n* = 2 independent experiments). Bar diagrams show the statistical evaluation. **(d)** RNA sequencing was performed to determine the expression of genes encoding G_s_-coupled receptors or orphan receptors in HUVECs and MLEC. Shown is a histogram of the library normalized counts. **(e)** HUVECs were transfected with control siRNA or siRNA directed against *GNAS* or the indicated GPCR RNAs, and HUVEC proliferation was determined after 24 h of culture in the absence or presence of GFP-expressing MeWo tumor cells by staining for Ki67 (*n* = 3). **(f)** Whole mount retinae of control and EC-Calcrl-KO mice at P7 stained with Isolectin B4. The bar diagram shows the statistical valuation of the quantification of the indicated vasculature parameters (*n* = 4 for control and *n* = 5 for KO mice). **(g)** LLC1 tumor cells were injected subcutaneously into wild-type and EC-Calcrl-KO mice, and tumor growth was determined (*n* = 6 mice per group). Bar length: 100 µm (a–c); 500 μm, 100 μm (f). Data represent mean values ± SEM; *, P ≤ 0.05; **, P ≤ 0.01; ***, P ≤ 0.001; n.s., non-significant (two-way ANOVA and Bonferroni’s post hoc test [a and g] and two-tailed Student’s *t* test [b, c, and f]).

To understand the role of endothelial Gα_s_ in tumor progression, we performed an siRNA-mediated knock-down of Gα_s_ in HUVECs and cultured the cells with or without GFP-expressing MeWo human melanoma or MDA-MB-231 human breast cancer cells. Gα_s_ knock-down in HUVECs did not have a significant effect on the proliferation of endothelial cells grown in the absence of tumor cells (Fig. 2 a and Fig. S2 a). However, when HUVECs were cultured together with MeWo or MDA-MB-231 cells, the number of proliferating endothelial cells increased, and this effect was lost after siRNA-mediated suppression of endothelial expression of Gα_s_ (Fig. 2 a and Fig. S2 a). Interestingly, knock-down of Gα_s_ also strongly reduced tumor cell proliferation in this co-culture model (Fig. 2, b and c; and Fig. S2 b), an effect which was not due to a change in the rate of apoptosis of endothelial cells or tumor cells (Fig. 2 d and Fig. S2 c). These results indicate that endothelial G_s_-mediated signaling promotes endothelial cell proliferation only in the presence of tumor cells and that it also increases proliferation of tumor cells.

**Figure 3.**
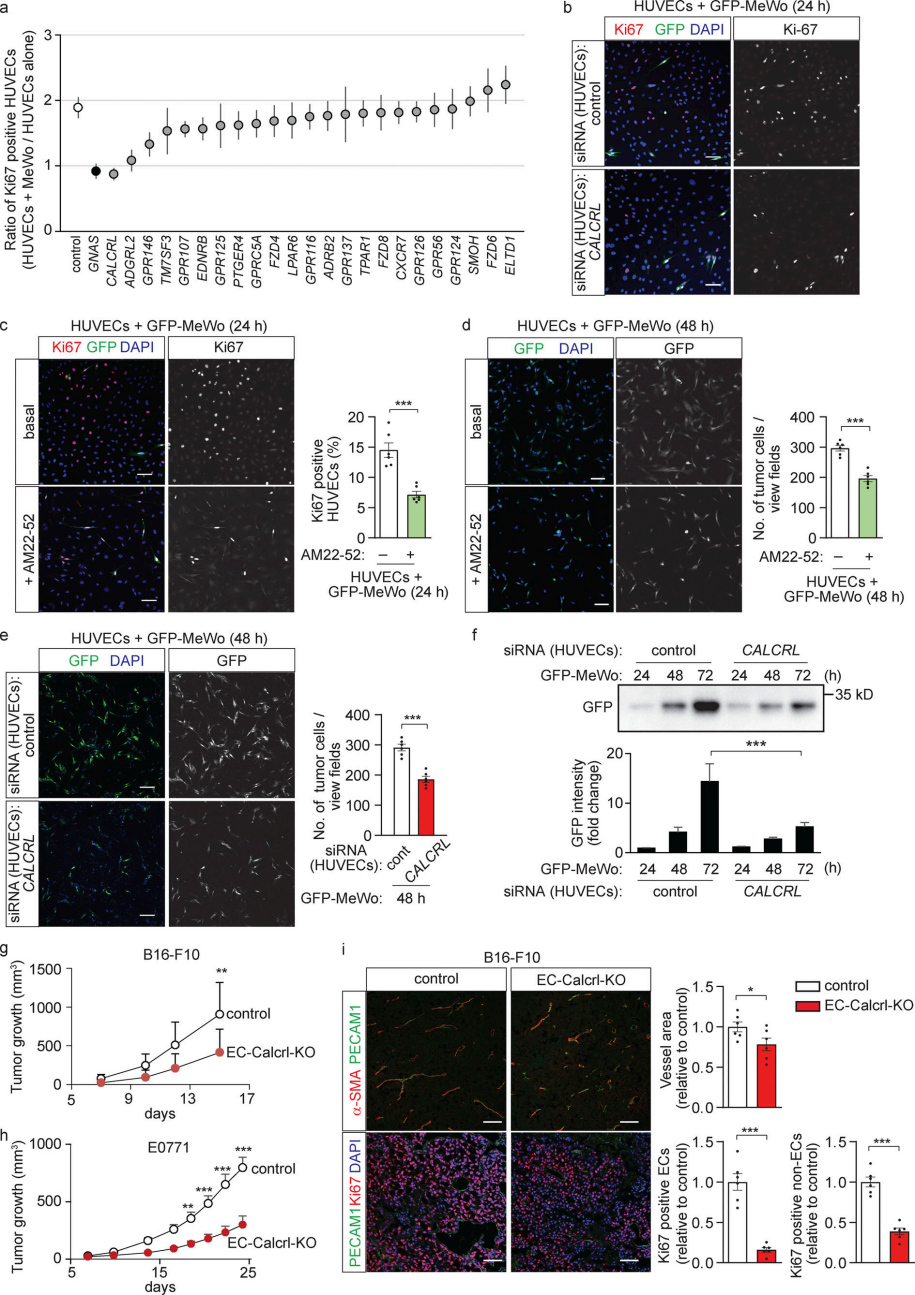
**The G**_**s**_**-coupled adrenomedullin receptor CALCRL is required for endothelial and tumor cell proliferation in vitro and in vivo. (a and b)** HUVECs were transfected with control siRNA or siRNA directed against *GNAS* or the indicated GPCR RNAs, and HUVEC proliferation in the absence and presence of GFP-MeWo tumor cells was determined by staining for Ki67. Shown are the ranked average ratios of Ki67-positive HUVECs co-cultured with MeWo cells and Ki67-positive HUVECs grown alone (a) and representative immunofluorescence staining (b; *n* = 4 independently evaluated dates). **(c–f)** HUVECs transfected with control siRNA or siRNA directed against *CALCRL* (e and f) or pretreated without or with 1 µM of the adrenomedullin receptor antagonist AM22-52 (c and d) were cultured with GFP-MeWo for the indicated time periods. Thereafter, Ki67-positive HUVECs were determined by staining with anti-Ki67 antibody (red) with DAPI (blue; c, *n* = 3 independent experiments), the number of GFP-expressing tumor cells was determined by either immunofluorescence (d and e, *n* = 3 independent experiments), or the expression level of GFP was determined by Western blot analysis (f). The bar diagrams show the statistical evaluation or represent the relative densitometric values based on Fiji software (f; *n* = 3 independent experiments). **(g)** B16-F10 melanoma cells were injected subcutaneously in control or EC-Calcrl-KO mice, and tumor growth was determined (*n* = 7 mice for each genotype). **(h)** E0771 breast cancer cells were injected into mammary fat pad of control or EC-Calcrl-KO mice, and tumor growth was determined (*n* = 5 mice for each genotype). **(i)** Immunohistochemistry of B16-F10 tumors grown in control or EC-Calcrl-KO mice. Sections were analyzed for markers of endothelial cells (PECAM1; green), perivascular cells (α-SMA; red) or proliferating cells (Ki67; red), and were stained with DAPI (blue). Bar diagrams show the statistical evaluation (*n* = 6 mice for each genotype). Bar length: 100 μm (b–e and i). Data represent mean values ± SEM; **, P ≤ 0.01; ***, P ≤ 0.001 (two-way ANOVA and Bonferroni’s post hoc test [f–h], two-tailed Student’s *t* test [c–e and i]).

To identify the receptor operating upstream of G_s_ in endothelial cells, we performed an siRNA-mediated knock-down of 23 G_s_-coupled receptors or orphan receptors which are highly expressed in HUVECs and primary mouse lung endothelial cells (MLECs; Fig. S2 d), and determined endothelial cell proliferation in the absence or presence of MeWo melanoma cells (Fig. 3 a and Fig. S2 e). While suppression of the majority of the candidate receptors had no effect on MeWo-dependent endothelial proliferation, knock-down of the CALCRL as well as of the adhesion GPCR latrophilin-2 (ADGRL2) inhibited MeWo-induced proliferation of endothelial cells to a similar degree as knock-down of Gα_s_ (Fig. 3 a). Since knock-down of ADGRL2 also reduced basal proliferation of HUVECs in the absence of MeWo cells (Fig. S2 e), we focused on CALCRL, which functions as a receptor for adrenomedullin (Poyner et al., 2002). Similar to knock-down of endothelial Gα_s_, loss of endothelial CALCRL or blockade of CALCRL by the competitive adrenomedullin receptor antagonist adrenomedullin 22-52 (AM22-52) reduced not only the tumor cell–induced growth of endothelial cells (Fig. 3, b and c) but also tumor cell proliferation in the co-culture model (Fig. 3, d–f).

To test the role of endothelial CALCRL in vivo, we analyzed tamoxifen-inducible endothelium-specific CALCRL-deficient mice (Tek-CreERT2;*Calcrl*^flox/flox^, herein referred to as EC-Calcrl-KO mice; Iring et al., 2019). Similar to EC-Gα_s_-KO mice, loss of endothelial CALCRL had no effect on retinal vasculature development (Fig. S2 f). However, tumor growth of subcutaneously injected B16-F10 and LLC1 cells as well as orthotopically injected E0771 breast cancer cells was significantly decreased in EC-Calcrl-KO mice compared to control animals (Fig. 3, g and h; and Fig. S2 g). Tumors in EC-Calcrl-KO mice showed reduced endothelial and non-endothelial cell proliferation as well as reduced vessel density compared to control animals (Fig. 3 i). These data indicate that the G_s_-coupled adrenomedullin receptor on endothelial cells mediates endothelial and tumor cell proliferation in vitro and in vivo.

### Tumor cell–derived adrenomedullin promotes endothelial and tumor cell proliferation

**Figure 4.**
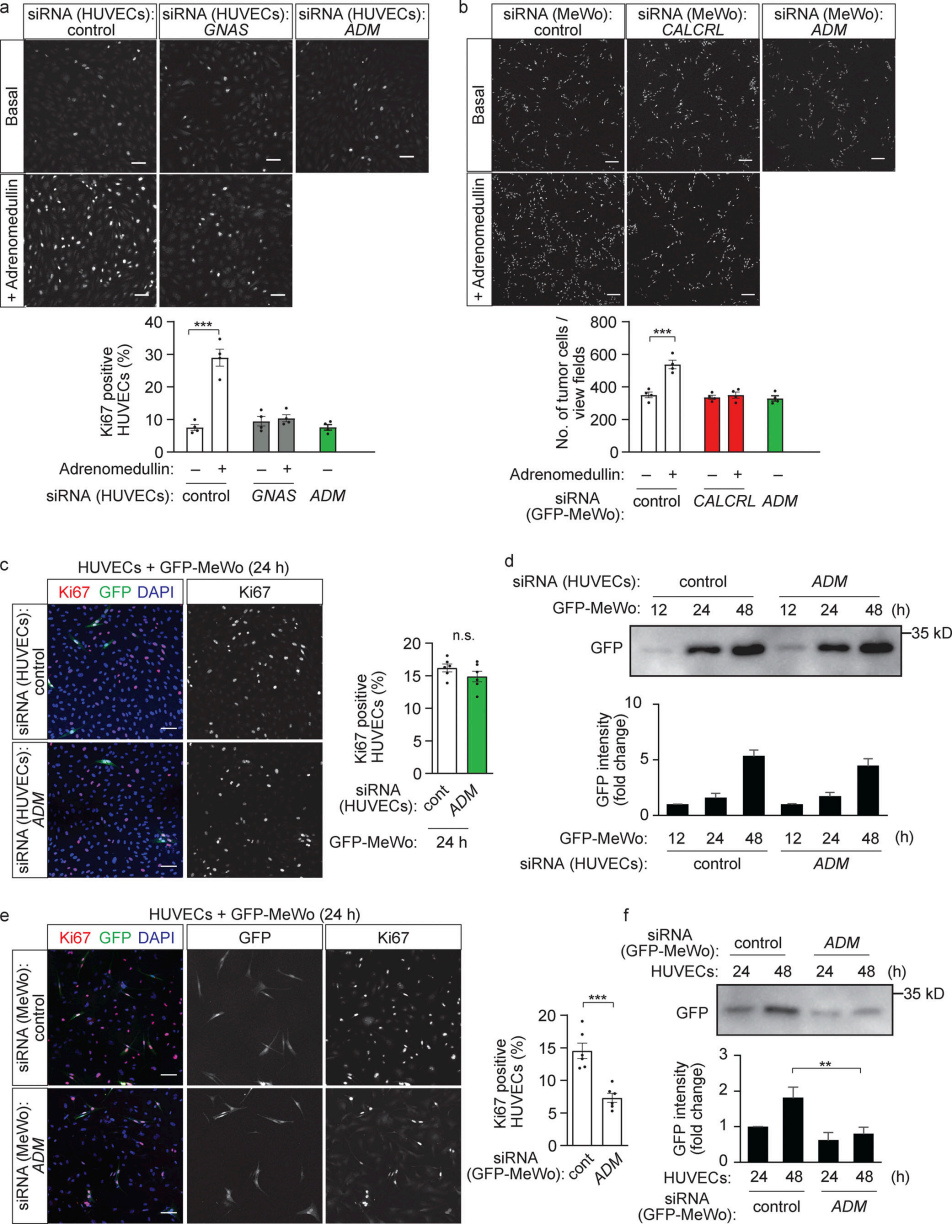
**Tumor cell–derived adrenomedullin promotes endothelial and tumor cell proliferation in vitro. (a)** HUVECs transfected with control siRNA or siRNA directed against *GNAS* or *ADM* were cultured without (basal) or with 10 nM adrenomedullin for 24 h and were then stained with an anti-Ki67 antibody. The bar diagram shows the statistical evaluation. **(b)** GFP-MeWo cells were transfected with control siRNA or siRNA directed against *CALCRL* or *ADM* and were cultured without (basal) or with 2 nM adrenomedullin for 24 h. Thereafter, the number of GFP-expressing tumor cells was determined. The bar diagram shows the statistical evaluation (a and b; *n* = 2 independent experiments, two evaluated areas per experiments). **(c and d)** HUVECs were transfected with control siRNA or siRNA directed against *ADM* and were cultured with GFP-MeWo for the indicated time periods. Endothelial proliferation was determined by staining with anti-Ki67 antibody (c), or the expression level of GFP was determined by Western blot analysis as an indicator of tumor cell growth (d). **(e and f)** HUVECs were cultured with GFP-MeWo transfected with control siRNA or siRNA directed against *ADM* for the indicated time periods. Endothelial proliferation was determined by staining with anti-Ki67 antibody (e), or the tumor cell proliferation was determined by quantifying expression level of GFP by Western blot analysis (f). The bar diagrams show the statistical evaluation (c and e; *n* = 3 independent experiments) or represent the relative densitometric values based on Fiji software (d and f; *n* = 3 independent experiments). Bar length: 100 μm (a–c and e). Data represent mean values ± SEM; *, P ≤ 0.05; **, P ≤ 0.01; ***, P ≤ 0.001 (one-way ANOVA and Tukey’s post hoc test [a and b], two-way ANOVA and Bonferroni’s post hoc test [d and f], and two-tailed Students *t* test [c and e]).

**Figure S3.**
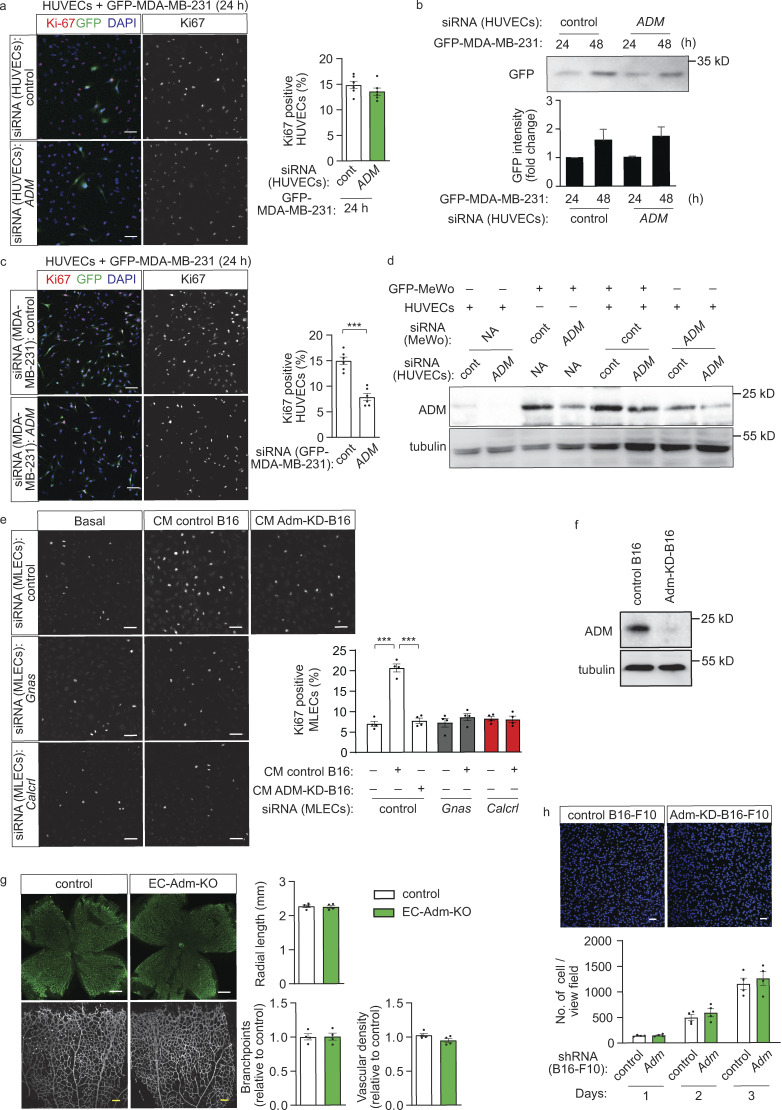
**Role of adrenomedullin in proliferation of endothelial and tumor cells. (a and b)** HUVECs transfected with control siRNA or siRNA directed against *ADM* were cultured with GFP-MDA 231 for the indicated time periods, then either stained with anti-Ki67 antibody (red) and with DAPI (blue; a) or lysed to analyze GFP expression as an indicator of tumor cell number (b; *n* = 3 independent experiments). **(c)** HUVECs were co-cultured for 24 h with GFP-expressing MDA-MB-231 cells transfected with control siRNA or siRNA directed against *ADM.* Endothelial proliferation was determined by analyzing immunofluorescence obtained with the anti-Ki67 antibody. Bar diagrams show the statistical evaluation (*n* = 3 independent experiments). **(d)** HUVECs transfected with control siRNA or siRNA directed against *Adm* were cultured alone or together with MeWo cells transfected with control siRNA or siRNA directed against adrenomedullin. Thereafter, adrenomedullin levels were determined by Western blot analysis. **(e)** MLECs were transfected with control siRNA or siRNA directed against *Gnas* or *Calcrl,* then cultured without (basal) or with conditioned medium (CM) of B16-F10 cells transfected with control shRNA (control B16) or shRNA directed against *Adm* (Adm-KD-B16). Endothelial proliferation was determined by staining with anti-Ki67 antibody (*n* = 2 independent experiments). **(f)** Expression of ADM in control B16-F10 melanoma cells and in Adm-KD-B16 was determined by Western blotting. **(g)** Whole mount retinae of control and EC-Adm-KO mice at P7 stained with Isolectin B4. The bar diagram shows the quantification of vasculature parameters (*n* = 4 for each genotype). **(h)** Control B16-F10 or Adm-KD-B16 cells were cultured for 72 h, and cells were counted. The bar diagram shows the statistical evaluation (*n* = 2 independent experiments). Bar length: 100 μm (a, c, e, and g); and in panel h: 50 μm. Data represent mean values ± SD; *, P ≤ 0.05; **, P ≤ 0.01; ***, P ≤ 0.001 (one-way ANOVA and Tukey’s post hoc test [e], two-way ANOVA and Bonferroni’s post hoc test [b and h] and two-tailed Student’s *t* test [a, c, and g]).

Adrenomedullin is expressed both in endothelial cells and tumor cells and can induce the proliferation of both endothelial cells and tumor cells (Hinson et al., 2000; Nikitenko et al., 2006) in a CALCRL- and Gα_s_-dependent manner (Fig. 4, a and b). To identify the major source of adrenomedullin responsible for the proliferation of endothelial and tumor cells in the co-culture model, we studied the effect of an adrenomedullin knock-down in endothelial or in tumor cells on the proliferation of both co-cultured cell types. Suppression of adrenomedullin expression in HUVECs had no effect on the proliferation of endothelial cells when cultured alone (Fig. 4 a) or when co-cultured with tumor cells (Fig. 4 c and Fig. S3 a). Also, tumor cell proliferation was unaffected by suppressed endothelial *ADM* expression (Fig. 4 d and Fig. S3 b). In contrast, suppression of adrenomedullin expression in tumor cells led to a strong reduction in the proliferation of HUVECs (Fig. 4 e and Fig. S3 c) and of co-cultured tumor cells (Fig. 4 f). However, loss of adrenomedullin expression had no effect on growth of tumor cells cultured alone (Fig. 4 b). In addition, suppression of adrenomedullin expression in MeWo melanoma cells, but not in HUVECs, led to a strong reduction in adrenomedullin levels in the co-culture (Fig. S3 d). These data indicate that it is primarily tumor cell–derived adrenomedullin that promotes endothelial and tumor cell proliferation in the co-culture model.

**Figure 5.**
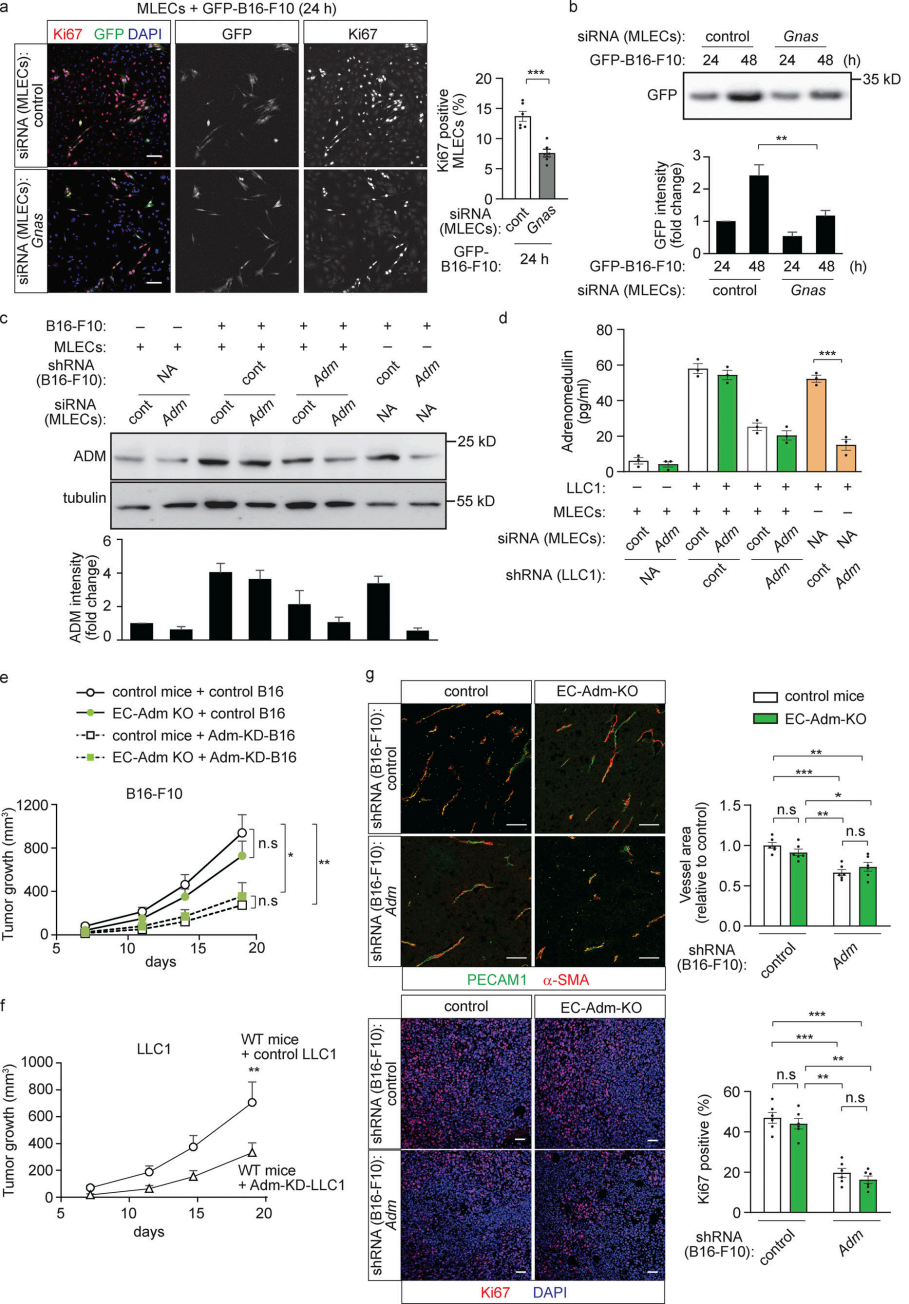
**Predominant role of tumor cell–derived adrenomedullin in tumor growth in vitro and in vivo. (a and b)** MLECs transfected with control siRNA or siRNA directed against *Gnas* were cultured with GFP-expressing B16-F10 cells (GFP-B16-F10) for the indicated time periods. Endothelial proliferation was determined by staining with an anti-Ki67 antibody (a), or the tumor cell growth was determined by measuring protein levels of GFP by Western blot analysis (b). The bar diagrams show the statistical evaluation (a; *n* = 3 independent experiments) or the relative densitometric values based on Fiji software (b; *n* = 3 independent experiments). **(c and d)** MLECs transfected with control siRNA or siRNA directed against *Adm* were cultured alone or together with B16-F10 cells (c) or LLC1 cells (d) transduced with scrambled control shRNA or shRNA directed against adrenomedullin. Thereafter adrenomedullin levels were determined by immunoblotting (c), or adrenomedullin concentrations in cell supernatant ware determined by ELISA (d). The bar diagrams in panel c show the relative densitometric values based on Fiji software (*n* = 3 independent experiments). **(e)** B16-F10 cells transduced with scrambled control shRNA (control B16) or shRNA directed against adrenomedullin (Adm-KD-B16) were injected subcutaneously in control or EC-Adm-KO mice, and tumor growth was determined (*n* = 6 mice for each condition). **(f)** LLC1 cells transduced with control shRNA (control LLC1) or shRNA directed against *Adm* (Adm KD LLC1) were injected subcutaneously in wild-type mice, and tumor growth was determined (*n* = 5 mice for each genotype). **(g)** Sections of tumors from control animals and EC-Adm-KO mice injected with control B16-F10 or Adm-KD-B16 cells were analyzed for markers of endothelial cells (PECAM1; green), perivascular cells (α-SMA; red) or for proliferating cells (Ki67; red), and were stained with DAPI (blue). The bar diagram shows the statistical analysis of the vessel area (f, *n* = 6 per mice group). Bar length: 100 μm (a), 50 μm (g). Data represent mean values ± SEM; *, P ≤ 0.05; **, P ≤ 0.01; ***, P ≤ 0.001 (two-way ANOVA and Bonferroni’s post hoc test [b and e–g], one-way ANOVA and Tukey’s post hoc test [c and d], and two-tailed Student’s *t* test [a]).

Similar observations were made in a murine in vitro co-culture model. When primary MLECs were cultured together with B16-F10 cells, the proliferation of endothelial and tumor cells was strongly reduced after knock-down of endothelial Gα_s_ (Fig. 5, a and b). In addition, suppression of adrenomedullin expression in tumor cells, but not in MLECs, led to reduced adrenomedullin expression and release in the co-culture (Fig. 5, c and d). Consistent with a role of tumor cell–derived adrenomedullin in endothelial and tumor cell proliferation under co-culture conditions, MLECs showed increased proliferation when exposed to conditioned medium of B16-F10 cells, an effect lost after knock-down of endothelial Gα_s_ and CALCRL (Fig. S3 e). However, conditioned medium from melanoma cells transduced with shRNA directed against adrenomedullin RNA (Adm-KD-B16; Fig. S3 f) did not increase endothelial proliferation (Fig. S3 e).

A predominant role of tumor cell–derived adrenomedullin was also seen under in vivo conditions. In mice with inducible endothelium-specific loss of adrenomedullin (Tek-CreERT2;*Adm*^flox/flox^ mice, herein referred to as EC-Adm-KO mice; Iring et al., 2019), both retinal angiogenesis as well as tumor angiogenesis and tumor growth were normal (Fig. 5, e and g; and Fig. S3 g). However, while loss of adrenomedullin expression in B16-F10 cells did not affect growth of melanoma cells when cultured alone in vitro (Fig. S3 h), it strongly reduced tumor growth in vivo (Fig. 5 E). This effect was not affected by loss of endothelial adrenomedullin (Fig. 5 e). Suppression of adrenomedullin expression in LLC1 tumor cells also resulted in reduced tumor growth in vivo (Fig. 5 f), and loss of tumor derived adrenomedullin, but not of endothelial adrenomedullin, reduced tumor angiogenesis and cell proliferation (Fig. 5 g). Together, these results indicate that tumor cell derived adrenomedullin promotes tumor growth through the endothelial CALCRL/G_s_ signaling pathway.

### Adrenomedullin inhibits endothelial CCL2 expression through its G_s_-coupled receptor

**Figure 6.**
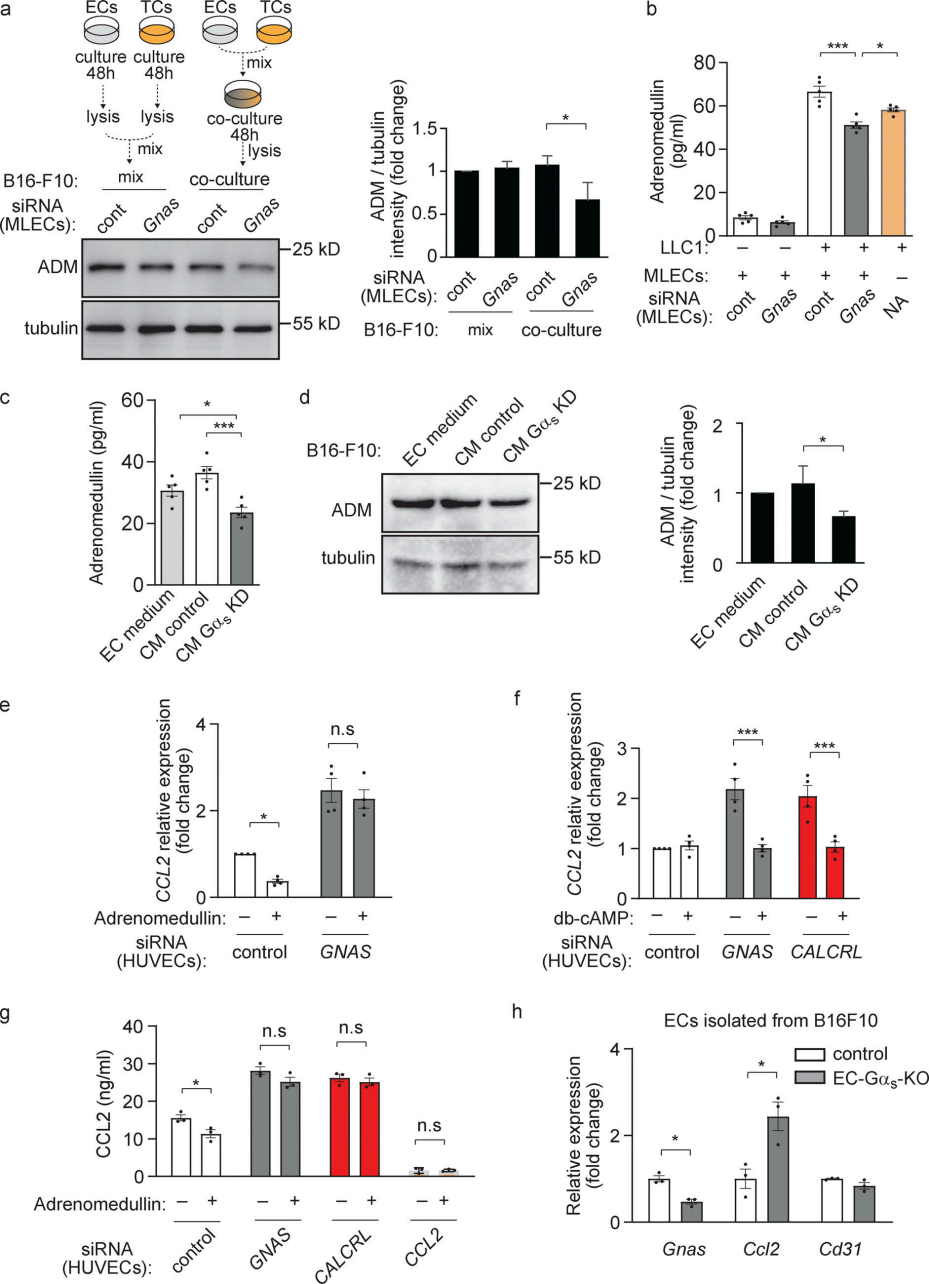
**Endothelial CCL2 was inhibited through an adrenomedullin/Gα**_**s**_**/cAMP signaling pathway. (a)** MLECs (ECs) transfected with control siRNA (cont) or with siRNA directed against *Gnas* were cultured with B16-F10 (TCs) for 48 h (co-culture, right) or were cultured alone for 48 h, lysed and then mixed with B16-F10 lysates (mix, left). The expression level of ADM in mixed lysates or lysates of co-cultures was analyzed by Western blotting. The bar diagram represents the relative densitometric values based on Fiji software (*n* = 3 independent experiments). **(b)** MLECs transfected with control siRNA (cont) or with siRNA directed against *Gnas* were cultured alone or together with LLC1 cells for 24 h, and ADM protein concentrations in cell supernatants was determined by ELISA (*n* = 5 independent experiments). **(c and d)** B16-F10 cells were cultured for 24 h with endothelial cell (EC) medium or with conditioned endothelial cell medium (CM) of MLECs transfected with control siRNA (CM control) or siRNA directed against *Gnas* (CM Gα_s_ KD). ADM protein concentrations in cell supernatants were determined by ELISA (c; *n* = 5 independent experiments), or the expression level of ADM was determined by Western blot (d; *n* = 3 independent experiments). **(e and f)** HUVECs transfected with control siRNA or siRNA directed against *GNAS* or *CALCRL* were incubated without or with 10 nM of adrenomedullin for 3 h (e), or with 50 µM db-cAMP for 24 h (f). Thereafter, the *CCL2* expression was determined by qRT-PCR analysis (*n* = 4 independent experiments). **(g)** HUVECs transfected with control siRNA or siRNA directed against *GNAS* or *CALCRL* or *CCL2* were incubated without or with 10 nM adrenomedullin for 24 h, and the CCL2 concentration in cell supernatants was determined (*n* = 3 independent experiments). **(h)** Endothelial cells were isolated from B16-F10 tumors grown in control or EC-Gα_s_-KO mice by FACS, and expression of the indicated genes was determined by qRT-PCR (*n* = 3 mice for each genotype). Data represent mean values ± SEM; *, P ≤ 0.05; **, P ≤ 0.01; ***, P ≤ 0.001; n.s., non-significant (two-way ANOVA and Bonferroni’s post hoc test [a and e–g], one-way ANOVA and Tukey’s post hoc test [b–d], and two-tailed Student’s *t* test [h]).

**Figure S4.**
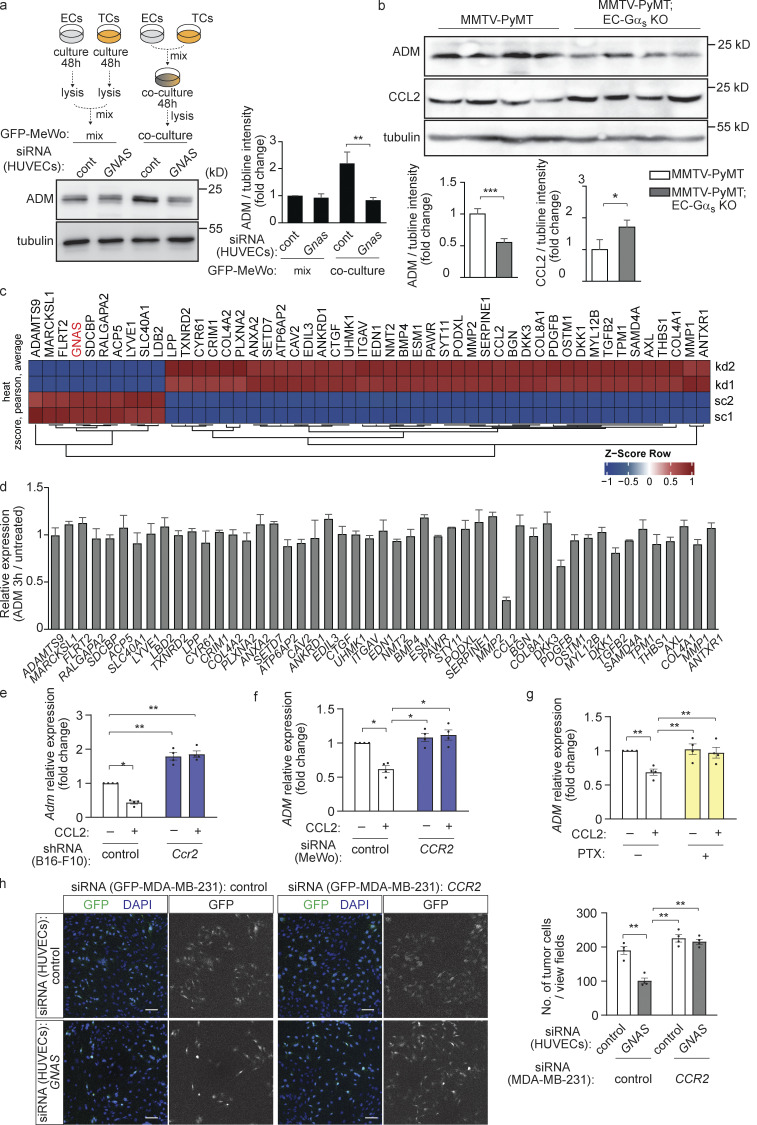
**Role of CCL2 in the interaction of tumor cells and endothelial cells. (a)** HUVECs (ECs) transfected with control siRNA (cont) or siRNA directed against *GNAS* were cultured with GFP-MeWo (TCs) for 48 h (co-culture, right) or cultured alone, lysed, and then mixed with GFP-MeWo lysates (mix, left). The expression level of ADM and Gα_s_ were analyzed by Western blotting. The bar diagram represents the relative densitometric values of the band recognized by the anti-ADM antibody based on Fiji software (*n* = 3 independent experiments). **(b)** Western blot analysis of mammary gland lysates dissected from MMTV-PyMT mice crossed with control or EC-Gα_s_-KO mice showing ADM and CCL2 protein levels as well as tubulin levels as loading control. **(c)** Heat map showing top 50 differentially expressed genes (based on false discovery rate) that significantly have increased (red) or decreased (blue) expression following *GNAS* silencing (kd) in HUVECs compared to scrambled (sc) siRNA control. **(d)** HUVECs were incubated without or with 10 nM adrenomedullin for 3 h, and the expression of the indicated genes was determined by qRT-PCR analysis (*n* = 3). **(e and f)** B16-F10 cells were stably transduced with control shRNA or shRNA directed against *Ccr2* (e) or GFP-MeWo cells were transfected with control siRNA or siRNA directed against *CCR2* (f), and were then incubated without or with 50 ng/ml CCL2 for 3 h. Thereafter, the adrenomedullin gene expression was determined by qRT-PCR analysis (*n* = 4 independent experiments). **(g)** GFP-MeWo cells were incubated without or with 100 ng/ml pertussis toxin (PTX) overnight then incubated with 50 ng/ml CCL2 for 3 h. Thereafter, *ADM* expression was determined by qRT-PCR analysis. **(h)** HUVECs were transfected with scrambled control siRNA or siRNA directed against *GNAS* and were then cultured for 48 h together with GFP-MDA-MB-231 cells transfected with control siRNA or siRNA directed against *CCR2*. Thereafter, the number of GFP-expressing tumor cells was determined by immunofluorescence. The bar diagram shows the statistical evaluation (*n* = 2 independent experiments). Bar length: 100 μm (h). Data represent mean values ± SEM; *, P ≤ 0.05; **, P ≤ 0.01; ***, P ≤ 0.001 (two-way ANOVA and Bonferroni’s post hoc test [a and e–h] and two-tailed Student’s *t* test [b]).

The fact that loss of adrenomedullin expression in tumor cells affected tumor cell growth only in co-culture with endothelial cells (Fig. 4, b and f; and Fig. S3 h) suggested that endothelial cells mediate regulation of tumor cell proliferation by tumor cell–derived adrenomedullin. Consistent with this, knock-down of Gα_s_ in endothelial cells reduced expression and release of adrenomedullin in a co-culture system (Fig. 6, a and b; and Fig. S4 a). Similarly, tumors from MMTV-PyMT mice crossed with EC-Gα_s_-KO mice expressed significantly less adrenomedullin compared to tumors from normal MMTV-PyMT animals (Fig. S4 b). To test whether G_s_-mediated signaling in endothelial cells regulates the release of a diffusible endothelial factor, which in turn controls the formation of adrenomedullin in tumor cells, we cultured tumor cells in the presence of supernatants from endothelial cells with or without loss of Gα_s_. Whereas supernatants of control endothelial cells had no effect on the amount of adrenomedullin in tumor cells as well as on the release of adrenomedullin from tumor cells, supernatants from endothelial cells with suppressed Gα_s_ expression inhibited both expression and release of adrenomedullin (Fig. 6, c and d). This strongly indicates that G_s_-mediated signaling in endothelial cells regulates the formation and/or release of adrenomedullin from tumor cells through a diffusible factor.

To search for potential factors controlled by G_s_-mediated signaling in endothelial cells, we compared the transcriptome of control HUVECs and of HUVECs lacking Gα_s_. We found various genes whose expression was strongly up- or down-regulated after suppression of *GNAS*, the gene encoding Gα_s_ (Fig. S4 c). When we tested the effect of adrenomedullin on the expression of these genes, we found that expression of *CCL2* was strongly altered (Fig. S4 d). Expression of *CCL2*, which encodes a chemokine belonging to the CC chemokine family, increased after knock-down of Gα_s_ but was reduced in control cells exposed to adrenomedullin (Fig. 6 e). Knock-down of CALCRL also increased *CCL2* expression (Fig. 6 f), and incubation of cells with dibutyryl-cAMP, a membrane-permeable stable derivative of cAMP mimicking Gα_s_ activation, suppressed *CCL2* expression induced by knock-down of CALCRL or Gα_s_ (Fig. 6 f). The increased endothelial expression of *CCL2* after inhibition of CALCRL/G_s_-mediated signaling was accompanied by an increased CCL2 level in cellular supernatants (Fig. 6 g). Furthermore, *Ccl2* expression in tumor ECs isolated from EC-Gα_s_-KO mice was higher compared to control mice (Fig. 6 h), and tumors from MMTV-PyMT mice crossed with EC-Gα_s_-KO animals also expressed significantly more *Ccl2* compared to tumors without loss of endothelial Gα_s_ (Fig. S4 b). These results show that *CCL2* expression in ECs is inhibited by adrenomedullin acting through its G_s_-coupled receptor and subsequent cAMP signaling.

### Endothelial CCL2 inhibits expression of adrenomedullin by tumor cells

**Figure 7.**
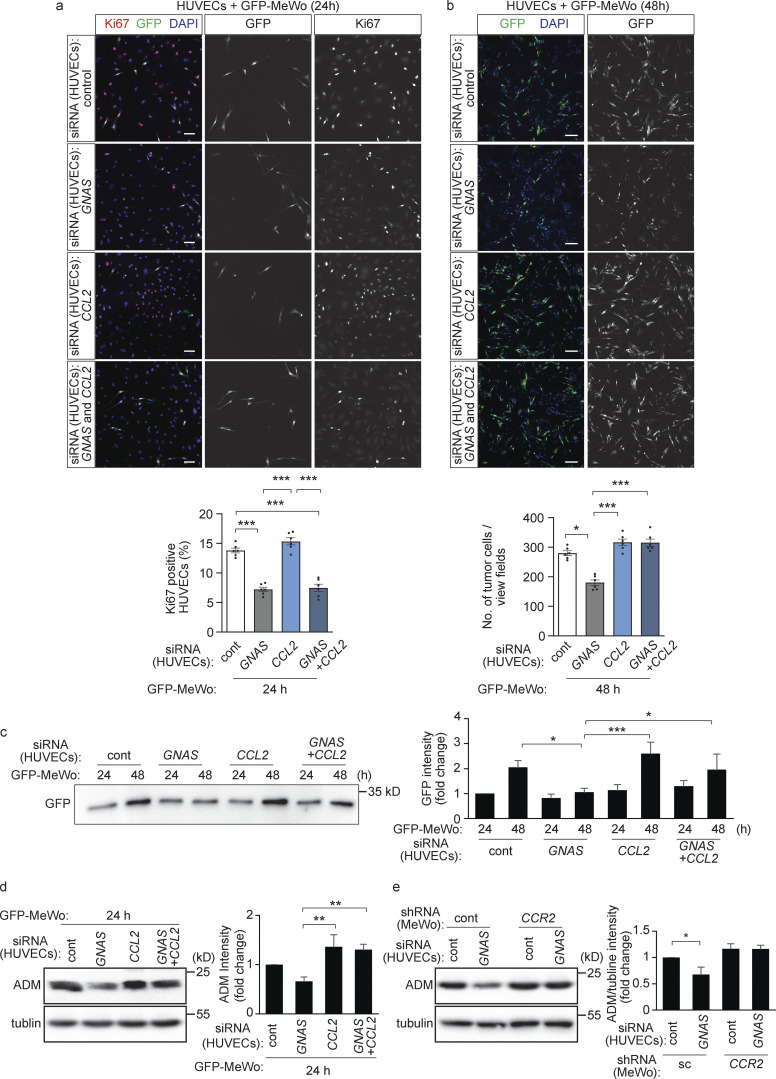
**Endothelial CCL2 regulates adrenomedullin expression and release in tumor cells in vitro. (a–d)** HUVECs were transfected with scrambled control siRNA or siRNA directed against *GNAS* and/or *CCL2* and were cultured with GFP-MeWo for the indicated time periods. Endothelial cell proliferation was determined by staining for Ki67 (a), and the number of GFP-expressing tumor cells was determined by immunofluorescence (b) or determined as the protein level of GFP by Western blot analysis (c). In addition, expression of *ADM* was analyzed by immunoblotting (d). The bar diagrams show the statistical evaluation (a and b; *n* = 3 independent experiments) or the relative densitometric values based on Fiji software (c and d; *n* = 3 independent experiments). **(e)** HUVECs were transfected with control siRNA or siRNA directed against *GNAS* and cells were co-cultured for 24 h with MeWo cells transfected with control siRNA or siRNA directed against *CCR2*. Thereafter, the expression level of ADM was determined by Western blot analysis. The bar diagrams represent the relative densitometric values of the band recognized by the anti-ADM antibody based on Fiji software (*n* = 3 independent experiments). Bar length: 100 μm (a and b). Data represent mean values ± SEM; *, P ≤ 0.05; **, P ≤ 0.01; ***, P ≤ 0.001 (two-way ANOVA and Bonferroni’s post hoc test [c and e], and one-way ANOVA and Tukey’s post hoc test [a, b, and d]).

To test whether CCL2 inhibits adrenomedullin formation in tumor cells, we incubated different tumor cells with CCL2. CCL2 indeed induced an inhibition of adrenomedullin expression, which was not seen after knock-down of its receptor CCR2 (Fig. S4, e and f). The effect of CCL2 on adrenomedullin expression was blocked by preincubation of cells with pertussis toxin indicating involvement of G_i_-type G-proteins (Fig. S4 g). We then analyzed the effect of endothelial loss of CCL2 on tumor cells and endothelial cells in the co-culture model. Knock-down of CCL2 in HUVECs with suppressed Gα_s_ expression had no effect on endothelial cell proliferation (Fig. 7 a) but reverted the effect of the Gα_s_ knock-down on tumor cell proliferation (Fig. 7, b and c) as well as on adrenomedullin levels (Fig. 7 d). In addition, the reduced adrenomedullin expression and proliferation of control tumor cells cultured with endothelial cells lacking Gα_s_ were normalized in tumor cells lacking CCR2 (Fig. 7 e and Fig. S4 h).

**Figure 8.**
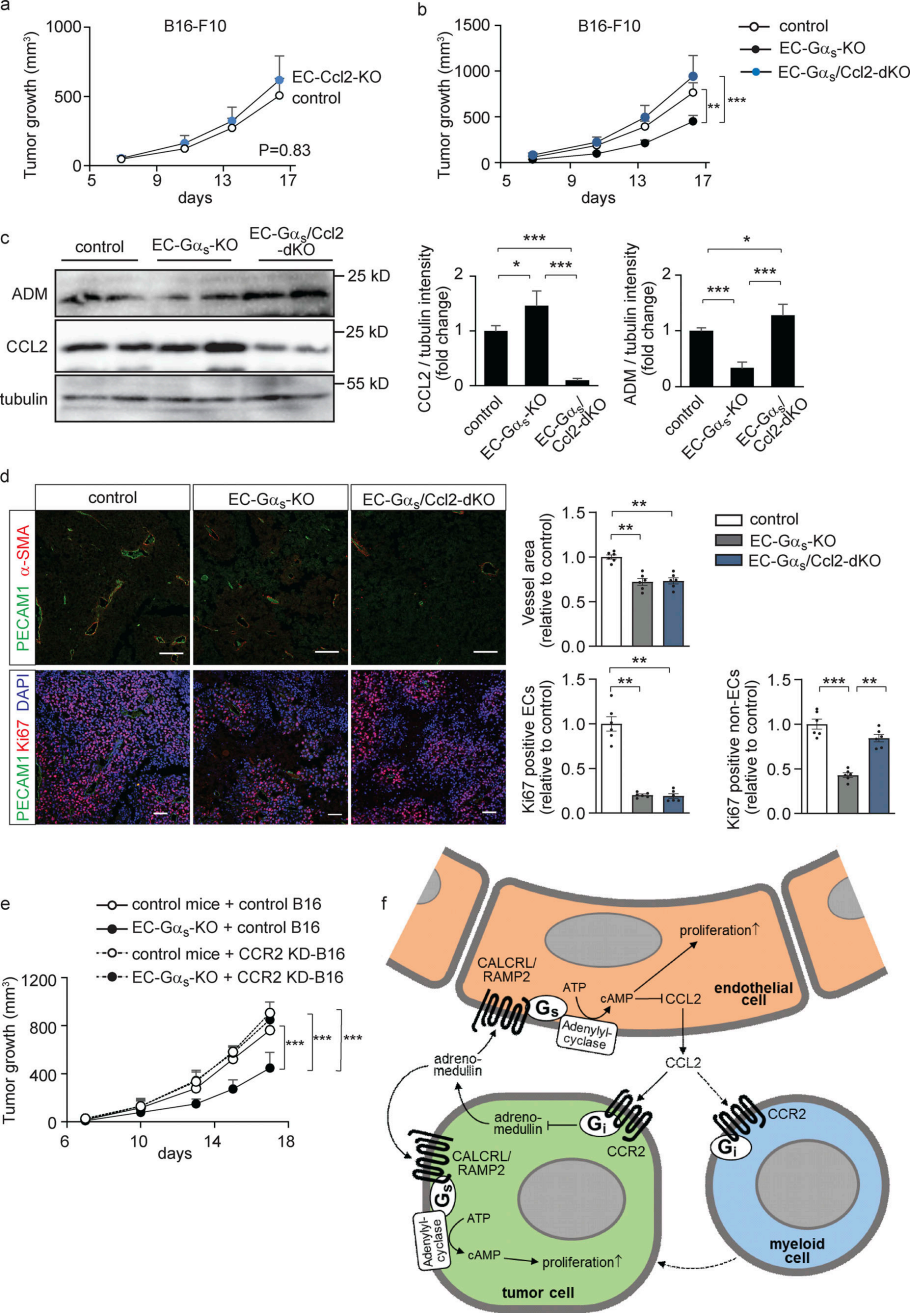
**Endothelial CCL2 functions as a regulator of adrenomedullin expression by tumor cells in vivo. (a and b)** 1.25 × 10^5^ (a; *n* = 5 mice per group) or 2.5 × 10^5^ (b; *n* = 6 mice per group) B16-F10 tumor cells were injected subcutaneously in control animals (a and b), EC-Ccl2-KO (a), EC-Gα_s_-KO (b), or EC-Gα_s_/Ccl2-dKO (b) mice, and tumor growth was determined. **(c)** Western blot analysis of mammary gland lysates dissected from control, EC-Gα_s_-KO or EC-Gα_s_/Ccl2-dKO mice showing protein levels of ADM and CCL2. Determination of tubulin protein levels served as a loading control. **(d)** Immunohistochemistry of B16-F10 tumors dissected from control, EC-Gα_s_-KO, or EC-Gα_s_/Ccl2-dKO mice. Sections were analyzed for markers of endothelial cells (PECAM1; green), perivascular cells (α-SMA; red) or proliferating cells (Ki67; red) and were stained with DAPI (blue). The bar diagrams show the statistical evaluation (*n* = 6). **(e)** B16-F10 cells transduced with scrambled control shRNA (control B16) or shRNA directed against CCR2 (CCR2-KD-B16) were injected subcutaneously in control or EC-Gα_s_-KO mice, and tumor growth was determined (*n* = 5 mice for each condition). **(f)** Schematic representation showing the angiocrine role of CCL2 in the regulation of adrenomedullin expression by tumor cells and the effects of tumor cell–derived adrenomedullin on the proliferation of tumor and endothelial cells as well as endothelial CCL2 expression. Dashed lines indicate potential additional mechanisms of tumor growth regulation through CCL2. Bar length: 50 μm (d). Shown are mean values ± SEM; *, P ≤ 0.05; **, P ≤ 0.01; ***, P ≤ 0.001 (two-way ANOVA and Bonferroni’s post hoc test [a, b, and e], and one-way ANOVA and Tukey’s post hoc test two-tailed Student’s *t* test [c and d]).

Finally, we analyzed the role of endothelial CCL2 in tumor progression under in vivo conditions. Endothelium-specific CCL2-deficient mice (Tek-CreERT2;*Ccl2*^flox/flox^ mice, herein referred to as EC-Ccl2-KO mice) did not show any differences in tumor growth when injected with B16-F10 melanoma cells (Fig. 8 a). However, when crossed with EC-Gα_s_-KO mice to generate inducible endothelium-specific Gα_s_/CCL2 double-deficient mice (EC-Gα_s_/Ccl2-dKO) we found that loss of endothelial CCL2 normalized the tumor phenotype of EC-Gα_s_-KO animals (Fig. 8 b). Tumors from EC-Gα_s_-KO mice showed significantly higher CCL2 levels and reduced *Adm* expression compared to tumors of control mice (Fig. 8 c). However, tumors of EC-Gα_s_/Ccl2-KO mice had normalized adrenomedullin expression compared to EC-Gα_s_-KO animals (Fig. 8 c). In contrast to EC-Gα_s_-KO mice, EC-Gα_s_/Ccl2-dKO animals showed increased tumor cell proliferation, whereas tumor vasculature was hardly affected (Fig. 8 d). In addition, suppression of CCR2 expression in tumor cells normalized tumor growth in EC-Gα_s_ KO animals, indicating that tumor cells are the main effector of endothelial CCL2 (Fig. 8 e). These data indicate that endothelial CCL2 can inhibit adrenomedullin formation but that this in turn is suppressed by adrenomedullin itself, which through its G_s_-coupled receptor on endothelial cells attenuates CCL2 formation.

## Discussion

Since endothelial cells when exposed to adrenomedullin in culture show typical angiogenic responses and, since overexpression of adrenomedullin in tumors or systemic application of adrenomedullin results in increased vascular and lymphatic angiogenesis (Iimuro et al., 2004; Karpinich et al., 2013; Martinez et al., 2002; Oehler et al., 2002; Zhang et al., 2017), adrenomedullin has been suggested to mediate tumor angiogenesis. However, evidence for a direct pro-angiogenic effect of adrenomedullin in tumors is still missing. Here, we show that loss of the adrenomedullin receptor or of the G-protein G_s_ in endothelial cells results in strongly reduced tumor angiogenesis in different syngeneic tumor models as well as in a genetic cancer model. In addition, our data revealed a novel mechanism by which adrenomedullin promotes its formation by tumor cells. In addition to its pro-angiogenic effect, adrenomedullin also inhibits endothelial formation of CCL2, which acts as an angiocrine factor and suppresses formation of adrenomedullin by tumor cells (Fig. 8 f). This confirms the role of adrenomedullin as a pro-angiogenic factor which also directly promotes tumor growth. Other pro-angiogenic factors such as VEGF and FGF can also affect tumor cells. It remains to be explored to what degree these pro-angiogenic and tumor-promoting factors synergize in particular tumors.

Our data clearly show that the endothelial G_s_-coupled adrenomedullin receptor mediates pro-angiogenic effects. How G_s_-mediated signaling resulting in increased cAMP levels promotes tumor angiogenesis is unclear. Several other G_s_-coupled receptors have been shown to increase tumor angiogenesis (Balzan et al., 2012; Garg et al., 2017; Yang et al., 2013; Zahalka et al., 2017) and the cAMP-dependent protein kinase A (PKA) has been shown to be a critical regulator of tumor angiogenesis (Bir et al., 2012; Garg et al., 2017; Xue et al., 2018). During development PKA has been shown to prevent hypersprouting and to regulate the number of tip cells, an effect which involves inhibitory regulation of autophagy (Nedvetsky et al., 2016; Zhao et al., 2019). Another study has shown that the cAMP/PKA pathway can promote the release of VEGF (Garg et al., 2017) and, in prostate cancer endothelial cells, the G_s_-coupled β2 adrenergic receptor has been shown to induce an angiogenic switch, which occurs through the alteration of endothelial cell metabolism by inhibiting endothelial oxidative phosphorylation (Zahalka et al., 2017). In addition, it is also possible that G_s_/cAMP-mediated pro-angiogenic effects are mediated by the cAMP-activated Rap1 GTP exchange factor EPAC, which has been shown to promote endothelial responses during tumor angiogenesis (Chrzanowska-Wodnicka, 2017; Garg et al., 2017).

Whereas the loss of the adrenomedullin receptor as well as of G_s_ in endothelial cells reduced tumor angiogenesis, postnatal angiogenesis in the retina was not affected. This may underline the functional differences between tumor and developmental angiogenesis (Chung and Ferrara, 2011; Jin and Jakobsson, 2012; Papetti and Herman, 2002) and indicates that endothelial adrenomedullin signaling pathway is not involved in developmental retinal angiogenesis. In contrast to our data, it has been reported that an endothelium-specific knock-out of RAMP2, which together with CALCRL forms the adrenomedullin receptor, affects both retinal angiogenesis and tumor angiogenesis (Iesato et al., 2013; Tanaka et al., 2016). This difference is most likely due to the requirement of RAMP2 for modulation of the function of a different G-protein-coupled receptor. In fact, RAMP2 has been shown to interact with several other GPCRs, including GPR4 and GPR182 (Lorenzen et al., 2019; Serafin et al., 2020), which are expressed in endothelial cells (Jing et al., 2016; Kechele et al., 2017; Le Mercier et al., 2021; Wyder et al., 2011).

In addition to the pro-angiogenic effect, we found that adrenomedullin also suppressed the formation of endothelial CCL2 through cAMP-PKA, and thereby resulted in the suppression of CCL2-induced inhibition of adrenomedullin formation by tumor cells. This inhibitory regulation resulted in increased adrenomedullin signaling and a self-reenforcement of endothelial adrenomedullin-induced CCL2 inhibition. Adrenomedullin has been shown to inhibit CCL2 production in endothelial cells and other cell types (Huang et al., 2011; Iwamoto et al., 2003; Yuda et al., 2012). Since cAMP has been shown in endothelial cells to inhibit NF-κB–mediated transcriptional regulation (Nakayama et al., 2020; Ollivier et al., 1996) and since NF-κB is well known to play a central role in regulating CCL2 expression (Martin et al., 1997), this would be the most likely mechanism underlying adrenomedullin-induced suppression of CCL2 expression.

The chemokine CCL2 and its receptor CCR2 have been shown to play multiple roles in tumorigenesis and tumor cell metastasis (Borsig et al., 2014; Gschwandtner et al., 2019; Zhang et al., 2010). Various data show that enhanced levels of CCL2 in different tumors promote tumor progression and metastasis. This is due to different mechanisms including the ability of CCL2 to recruit and regulate tumor-associated macrophages, which promote tumor progression and metastasis in part through its immunosuppressive role in the tumor microenvironment (Fridlender et al., 2011; Kersten et al., 2017; Li et al., 2017; Yang et al., 2019). CCL2 has also been shown to promote tumor progression by acting on endothelial cells to stimulate tumor cell extravasation and metastasis (Hauselmann et al., 2016; Wolf et al., 2012) or by a direct effect on tumor growth (Wong et al., 2020). However, CCL2 has also been shown to decrease tumor cell growth and metastasis (Asano et al., 1996; Rollins and Sunday, 1991; Takahashi et al., 2009). Several reports showed that CCL2 can synergize with bacterial endotoxin to activate macrophages to become tumoricidal and to reduce tumor progression and metastasis (Huang et al., 1995; Nakashima et al., 1998; Singh et al., 1993). In addition, CCL2 can have antitumorigenic and antimetastatic activity depending on cellular context and cancer type by regulating neutrophil activity (Granot et al., 2011; Lavender et al., 2017). Our data show a new role of CCL2 as an angiocrine factor of the tumor microenvironment which suppresses tumor progression by directly acting through CCR2 expressed by tumor cells. However, this activity can be suppressed by tumor-derived adrenomedullin and potentially also by other mediators which activate G_s_-coupled receptors. Consistent with this, *CCL2* has been found to be one of the most strongly downregulated genes in endothelial cells of invasive breast cancer compared to endothelial cells of normal mammary vasculature (Parker et al., 2004).

In summary, our data demonstrate that adrenomedullin is an essential regulator of tumor angiogenesis which, by acting through its G_s_-coupled receptor on endothelial cells, promotes endothelial cell proliferation. In addition, we show that adrenomedullin inhibits the formation of the endothelial angiocrine factor CCL2 which, in turn, can suppress adrenomedullin formation by tumor cells. It remains to be evaluated whether promoting endothelial CCL2 formation in adrenomedullin-producing tumors is a strategy to reduce tumor growth.

## Materials and methods

### Reagents

Adrenomedullin and PTX were purchased from Sigma-Aldrich (A-2327, P7208), and dibutyryl-cAMP was purchased from Merck Chemicals GmbH (28745). Human AM22–52 was purchased from Bachem (H-4144). Human and mouse recombinant CCL2 were purchased from Bio-Techne GmbH (279-MC, 479-JE). Matrigel was purchased from Corning BV (356237).

### Cells

HUVECs and MLECs were purchased from Lonza. HUVECs were cultured with EGM-2 (Lonza) and MLECs were cultured with endothelial growth medium (EGM-2-MV, Lonza). Confluent cells at passage ≤6 were used in all experiments. MDA-MB-231-GFP cells were from AntiCancer. MeWo cells were from CLS, and E0771 were from tebu-bio. B16-F10 and LLC1 cells were from ATCC. All tumor cell lines were cultured in DMEM supplemented with 10% FBS, penicillin/streptomycin (100 units/ml), and glutamine (2 mM). Cells were tested negative for mycoplasma contamination before experiments.

### siRNA-mediated knockdown

Cells at 70% confluence were transfected with siRNAs using Opti-MEM (Thermo Fisher Scientific) and lipofectamine RNAiMAX (Invitrogen) according to the manufacturer’s instructions. SiRNAs used for the screen are described in Table S1. SiRNAs directed against human *CALCRL* (EHU003121), human *CCR2* (EHU109141), human *GPR125* (EHU085311), human *FZD6* (EHU074771), human *TM7SF3* (EHU049511), and murine *Calcrl* (EMU054511) were purchased from Sigma-Aldrich. The targeted sequences of other siRNAs were as follows: human *GNAS*: 5′-CUG​AUU​GAC​UGU​GCC​CAG​U-3′; human *ADM* 5′-GGA​TGC​CGC​CCG​CAT​CCG​AG-3′; murine *Gnas*: 5′-CUG​AUU​GAC​UGU​GCC​CAG​U-3′; murine *Adm* 5′-GCA​AUC​AGA​GCG​AAG​CCC​A-3′.

Knockdown efficiencies were determined by Western blotting or by quantitative RT-PCR (qRT-PCR; LightCycler480, Roche).

### Co-culture experiments

For Ki67 staining or TUNEL staining, 1.5 × 10^3^ GFP-expressing tumor cells were added onto the endothelial monolayer and cultured overnight. For Ki67 staining, cells were fixed for 10 min in 4% paraformaldehyde (PFA). After washing with PBS, cells were blocked and permeabilized in blocking buffer (0.3% Triton X-100 and 1% BSA in 1× PBS) at room temperature for 15 min. Thereafter, cells were incubated with primary antibody directed against Ki67 (Abcam) overnight at 4°C (dilution 1:100). After gentle washing with PBS (three times), cells were incubated with corresponding Alexa Fluor 594–conjugated secondary antibody (1:500; Invitrogen) together with DAPI (1 ng/ml; Invitrogen) for 1 h at room temperature.

For determination of GFP by immunofluorescence or Western blot analysis, and determination of ADM or CCL2 protein concentrations in cell supernatants, 1 × 10^5^ endothelial cells and 1 × 10^5^ tumor cells were cultured in the same well. For determination of GFP by immunofluorescence, cells were fixed for 10 min in 4% PFA and washed with PBS. After permeabilization in blocking buffer for 30 min as described above, cells were stained with DAPI for 1 h at room temperature.

### TUNEL assay

1.5 × 10^3^ GFP-expressing tumor cells were added onto the endothelial monolayer and cultured overnight. Cells were then fixed for 15 min in 4% PFA at room temperature. Apoptotic cells were detected using the Click-iT TUNEL Alexa Fluor 594 Imaging Assay (C10246; Invitrogen) following the manufacturer’s instructions.

### Determination of ADM and CCL2 level

Supernatants collected from endothelial cells and/or tumor cells were transferred to precooled tubes, and cellular debris was removed by centrifugation (20,000 *g*) for 10 min at 4°C. Adrenomedullin and CCL2 concentrations were determined with an adrenomedullin ELISA Kit (catalog #LS-F6083-1; BIOZOL Diagnostica Vertrieb GmbH) or an MCP-1/CCL2 Human Uncoated ELISA Kit (88-7399-22; Invitrogen) following the manufacturer’s instructions.

### Western blot analysis

To extract total protein from cells, radioimmunoprecipitation assay buffer supplemented with protease inhibitors (10 mg/ml of leupeptin, pepstatin A, 4-(2-aminoethyl)benzensulfonylfluorid and aprotinin), and phosphatase inhibitors (PhosSTOP, Roche) was used. To extract total protein from tumor, the tissue was placed in three volumes of ice-cold lysis buffer (20 mM Tris, 1 mM EDTA, 1 mM dithiothreitol, 0.1% SDS, 150 mM NaCl, 1% Triton-X with protease inhibitors) and homogenized. After centrifugation (20,000 *g*) for 10 min at 4°C, supernatant were transferred.

Sample lysates were subjected to SDS-PAGE and transferred to nitrocellulose or polyvinylidene difluoride membranes. After blocking (5% BSA or 0.3% skim milk in Tris-buffered saline [TBS] with 0.1 % Tween-20) at room temperature for 30 min), membranes were incubated with gentle agitation overnight at 4°C with the following primary antibodies: anti-GFP (3H9; Chromotek), anti-adrenomedullin (LS-B15534; LS Bio), anti-tubulin (2521; Cell Signaling), anti-Gα_s_ (sc-1359; Santa Cruz). The membranes were then washed three times for 7 min each with TBS with 0.1 % Tween-20 and incubated with HRP-conjugated secondary antibodies (dilution 1:3,000; Cell Signaling) followed by chemiluminescence detection using ECL substrate (Pierce) according to the manufacturer’s protocol. Band intensities from immunoblotting were quantified by densitometry using ImageJ software (Abramoff et al., 2004).

### Lentiviral infection of tumor cells

To generate tumor cell lines, in which expression of *Adm* or *Ccr2* was suppressed, the lentiviral pLV-Puro-U6 expression vector containing the sequence of scramble shRNA, murine Adm shRNA or murine Ccr2 shRNA (VectorBuilder) were used. To generate EGFP-expressing tumor cell lines pLV-Bsd-EF1A expression vector containing the sequence of EGFP (VectorBuilder, VB210426-1127uue) was used.

Each vector was transfected into HEK293T cells along with envelope plasmid pMD2.G and packaging plasmid psPAX2. Following 48 h of incubation, the supernatant was collected and filtered through a 0.45-μm low-protein binding Durapore membrane (Millex). Tumor cells were transduced for 24 h with the lentiviral vector, followed by a complete medium change. After 48 h, cells were used for further analyses.

### RNA sequencing

RNA was isolated from ECs using the miRNeasy Micro Kit (Qiagen). Samples were treated with on-column DNase digestion (DNase-Free DNase Set, Qiagen), and total RNA and library integrity were verified with LabChip Gx Touch 24 (PerkinElmer). 2 μg of total RNA were used as input for TruSeq Stranded mRNA Library preparation following the Low Sample protocol (Illumina). Sequencing was performed on a NextSeq500 instrument (Illumina) using v2 chemistry. The resulting raw reads were assessed for quality, adapter content, and duplication rates. Only reads between 30 and 150 nucleotides were cleared for further analyses. Trimmed and filtered reads were aligned versus the Ensembl human genome version hg38 (GRCh38).

### Quantitative real-time PCR analysis

Total RNA was isolated using the Quick-RNA Micro prep kit (Zymo) according to the manufacturer’s protocol. Quality control of samples was carried out using a Nanodrop ND-100 Spectrophotometer. Complementary DNA synthesis was performed using the ProtoScript II Reverse Transcription kit (M0368S; New England BioLabs). Quantitative real-time PCR was performed using primers designed with the online tool provided by Roche and the Light-Cycler 480 Probe Master System (Roche). Each reaction was run in duplicates, and relative gene expression levels were normalized to GAPDH. Relative expression was calculated using the ΔΔCt method. Primer sequences used are described in Table S2.

### Experimental mice

All mice were backcrossed onto a C57BL/6N background, and experiments were performed with littermates as controls. Mice were housed under a 12-h light/12-h dark cycle, with free access to food and water and under specific pathogen-free conditions unless stated otherwise. The generation of inducible endothelium-specific Gα_s_-deficient mice (Tie2-CreERT2;*Gnas*^flox/flox^), endothelium-specific CALCRL- and adrenomedullin-deficient mice (Tie2-CreERT2;*Calcrl*^flox/flox^ or Tie2-CreERT2;*Adm*^flox/flox^, respectively) was described previously (Iring et al., 2019). Mice carrying a floxed *Ccl2* allele were purchased from The Jackson Laboratory (stock no. 016849) and crossed with inducible endothelium-specific Cre transgenic mice (Tek-CreERT2; Korhonen et al., 2009). MMTV-PyMT mice were obtained from The Jackson Laboratory. Cre-mediated recombination in the adult mice was induced by i.p. injection of tamoxifen (T5648; Sigma-Aldrich) dissolved in Miglyol 812 (1 mg per mouse per day) on 5 consecutive days.

### Syngeneic tumor models

For syngeneic tumor experiments, age (8–10 wk) and sex-matched mice were used. 2.5 × 10^5^ tumor cells cultured at 75–80% confluency were suspended in 50 µl PBS, unless otherwise described, and were injected subcutaneously at the right flank. Mice were monitored every day, and tumor length (L) and width (w) were measured with a caliper. Tumor volume was calculated in the following way: 0.5 × (L × w^2^). Mice were sacrificed after 15 d and tumors were extracted and processed for immunostaining, Western blotting, or cell sorting.

For mammary fat pad injection, mice were anesthetized by subcutaneously injecting xylazin/ketamine (16 mg/kg, 120 mg/kg body weight respectively), or by isoflurane. After a small incision was made between the fourth nipple and the midline with a scissor, a pocket was formed by inserting a cotton swab moistened with PBS. Then the mammary fat pad was exposed using tweezers. 30 µl of E0771 cell suspension (10 million cells/ml) mixed with 20 µl cooled matrigel was injected into the mammary fat pad by holding the needle horizontally. Skin was sutured with absorbable 6.0 silk suture (CatGut). After surgery, animals were monitored until recovery in a chamber on a heating pad.

### MMTV-PyMT mice

Mice were monitored every day for tumor initiation and growth. All palpable masses were measured using external calipers, and tumor mass was calculated as described above. Mice remained in the study until the mass reached a total volume of 1.7 cm^3^ unless tumor ulceration or other complications occurred. Mice were also evaluated for their behaviors, grooming activities, body conditions and body weight and kept in the study until termination criteria were met. After reaching any of the previously described criteria, each mouse was euthanized with CO_2_ and tissue samples were collected for subsequent analyses.

### Analysis of retinal angiogenesis

Cre-mediated recombination was induced by i.p. injection of tamoxifen dissolved in Miglyol 812 (0.5 mg per mouse per day) on postnatal day 1 to 3. On day 7 or 27, whole eyes were fixed with 4% PFA for overnight at 4°C. After washing in PBS, retinae were dissected, cut into four quadrants and blocked/permeabilized (1% BSA, 0.3% Triton, PBS) overnight at 4°C. On the following day, retinae were washed two times in Pblec buffer (1% Triton X-100, 1 mM CaCl_2_, 1 mM MgCl_2_, and 1 mM MnCl_2_ in PBS [pH 6.8]) for 20 min and incubated with biotinylated isolectin-B4 (1:50; VectorLabs) for 2 h. Following five washes in blocking solution, retinae were incubated with Alexa Fluor streptavidin-conjugated antibodies (1:100; Molecular Probes) for 2 h, washed three times in blocking solution and were flat-mounted on microscope glass slides with Fluoromount-G (0100-01; SouthernBiotech). All images shown are representative of the retinal vascular phenotype observed in at least four individual pups. All quantifications were done with Fiji software on high-resolution confocal images or lower resolution stereomicroscope images (vascular outgrowth). The radial length was defined as the distance from the center of the retina to the angiogenic front for each retina quadrant. The vascular density was defined as the ratio of isolectin B4^+^ area/total area.

### Histology and immunostaining of tumors

Mice were sacrificed by CO_2_ inhalation, and the chest cavity was opened for perfusion with PBS. Tumors were dissected and fixed in 4% PFA overnight at 4°C and were maintained in 30% sucrose solution. Tissue embedding was performed in OCT-based sample blocks. Sample blocks were cryo-sectioned (5–7 μm thickness) using a cryostat. Sectioned tissues were permeabilized and blocked with 10% BSA and 0.1% Tween in TBS. They were then incubated with primary antibodies overnight at 4°C. Primary antibodies used were as follows: anti-PECAM1 (550274; BD Biosciences or ab24590; Abcam), anti-Ki67 (ab15580; Abcam), anti-α-SMA (F3777; Sigma-Aldrich), anti-cleaved caspase 3 (9661; Cell Signaling), anti-Hif1α (GTX30115; GeneTex), anti-CD68 (MCA1957; serotec) and anti-CD206 (565250; Bio-Rad). After washing three times with PBS, bound primary antibodies were detected using Alexa Fluor 488– or 594–conjugated secondary antibodies (1:500; Invitrogen). DAPI (1 ng/ml; Invitrogen) was used to label cell nuclei. Sections were viewed with a confocal microscope (Leica, SP5).

### Cell sorting and flow cytometric analysis

Mouse lung or tumors were dissected and enzymatically digested for 30 min while being shaken at 37°C in a digestion mix containing 0.25% collagenase II (Gibco) and 1 U/ml dispase (Gibco) in PBS. Cell suspensions were filtered through a 100-μm cell strainer followed by washing with 1% FCS in PBS. Antibodies used were as follows: CD31-PE (MCA2388PE; Serotec), CD45-APC (17-0451; eBioscience), CD4-PeCy7 (25-0041; eBioscience), CD8-PerCp-Cy5.5 (45-0081; eBioscience), CD45-FITC (553079; BD), CD19-PE (12-0193; eBioscience), Ly6G-APC (127613; Biolegend), F4/80-APC-efluor 780 (47-4801; eBioscience), CD206-PE (MCA2235PET; AbD Serotec), CD11c-FITC (557400; BD), CCR2-APC (150627; Biolegend). Cells were sorted on an S3e cell sorter (BioRad) or FACSCanto (BD).

### Study approval

All procedures of animal care and use in this study were approved by the local animal welfare authority committee of the Regierungspräsidium Darmstadt.

### Statistics

Trial experiments or experiments done previously were used to determine sample size with adequate statistical power. Samples were excluded in cases where RNA/cDNA quality or tissue quality after processing was poor (below commonly accepted standards). Animals were excluded from experiments if they showed any signs of sickness. The investigator was blinded to the group allocation and during the experiment. Data represent biological replicates. In all studies, comparison of mean values was conducted with unpaired, two-tailed Student’s *t* test or one-way or two-way ANOVA with Bonferroni’s post hoc test. In all analyses, statistical significance was determined at the 5% level (P < 0.05). Depicted are mean values ± SEM as indicated in the figure legends. Statistical analysis was performed with Prism5 or Prism6 (GraphPad) or Excel (Microscoft) software.

### Online supplemental material

Fig. S1 shows the effects of endothelial loss of Gα_s_ on retinal angiogenesis, tumor angiogenesis, and tumor microenvironments. Fig. S2 shows role of endothelial Gα_s_ or CALCRL, both in vitro and in vivo. Fig. S3 shows the effect of endothelial- or tumor cell–derived adrenomedullin on endothelial or tumor cell proliferation. Fig. S4 shows tumor-suppressing function of endothelial CCL2 whose formation is inhibited through adrenomedullin signaling. Table S1 shows target sequences of siRNAs used in the siRNA screen in Fig. 3 a and Fig. S2 b. Table S2 shows primer sequences used for qRT-PCR.

## Supplementary Material

Table S1 lists target sequences of siRNAs used in the siRNA screen.

Table S2 lists primer sequences used for qRT-PCR.
