# Supplementary material for: Suppression of CCL2 angiocrine function by adrenomedullin promotes tumor growth
Source: J Exp Med. 2022 Nov 14;220(1):e20211628. doi: 10.1084/jem.20211628 (PMC9665902; doi:10.1084/jem.20211628)
Supplement: Table S1 — lists target sequences of siRNAs used in the siRNA screen. [file JEM_20211628_TableS1.docx]

**Table S1.** Target sequences of siRNAs used in the siRNA screen

(Fig. 3 a and Fig S2 b)

| Gene | Target sequence (5′–3′) |
| --- | --- |
| ADGRL2 | GGAAUGGGCUUGCAAAGUU |
| ADGRL2 | GGCAAGAACUCAAUUGAUU |
| ADGRL2 | GAAAGAACGAGGAAUAUUG |
| ADGRL2 | CGACAAACGUGCCGCAUCA |
| ADRB2 | GCCATTACTTCACCTTTCA |
| CALCRL | CAUGACAGCUCAAUAUGAA |
| CALCRL | CAAAGUGUCCCAGUUCAUU |
| CALCRL | GUCAUGACUGUCCUAGUGA |
| CXCR7 | CCUACUACCUGAAGACCGU |
| EDNRB | GTTGGTATTGGACTATATT |
| EDNRB | AAGGAAGTTATCTGCGAAT |
| EDNRB | ATCGAG ATCAAGGAGACTT |
| ELTD1 | GGAAUUGAAGCCUGCUAUU |
| ELTD1 | CUCAAACCCACCCACAUUA |
| ELTD1 | GAAUAUUACAGAUUGUUCA |
| FZD4 | GAAAUGCACAGCUCUUAUU |
| FZD4 | GACAAAGACAGACAAGUUA |
| FZD4 | GAUCGAUUCUUCUAGGUUU |
| FZD4 | AGUCAAUCAUGUCGAGUCA |
| FZD8 | CUACAACCGCACCGACCUA |
| FZD8 | AGACAGGCCAGAUCGCUAA |
| FZD8 | UCACCGUGCCGCUGUGUAA |
| FZD8 | ACACCUACAUGCCCAAUCA |
| GPR107 | GAAAGAAUUGCCAAGUGA |
| GPR107 | GAACACCUUUGGCUUCUUC |
| GPR107 | AGACAAGGAUGUGACUAUU |
| GPR107 | GACGGAAUGAUGUAUUUAA |
| GPR116 | CACCAUUGCAGUUGCCUUA |
| GPR116 | GAAGCUAUGCCGGUUCUCA |
| GPR116 | GAAUACUCCUGGAUAUUAU |
| GPR124 | CGACUAAACAUAUCUGGAA |
| GPR124 | CAUUGGAGGCCUACCUCAU |
| GPR124 | CUCAUCACCUGGAUCUAUU |
| GPR126 | CAGAUAACAUUUAACGACU |
| GPR126 | GAACUUAACCUCAGCCAAU |
| GPR126 | GUGAUGAAUUCUGUUGGAU |
| GPR137 | GACGCTTATGAACCTCTACTT |
| GPR146 | AGCUAUUCAAUAGCAGUGACGCGCT |
| GPR56 | CUCCGAAGAGGCCCUCACA |
| GPR56 | GAAUGUGACUCUGCAAUGU |
| GPR56 | CCUUGGAUCUUGAGGGUCU |
| GPRC5A | AGGCAGCAUUUUUCGCCUGTT |
| LPAR6 | UCAGCAUGGUGUUUGUGCUUGGGUU |
| SMOH | CAACCUGUUUGCCAUGUUU |
| SMOH | GCUACAAGAACUACCGAUA |
| SMOH | UCGCUACCCUGCUGUUAUU |
| SMOH | GUGCCAACCUCUUUGCGUU |
| TPAR1 | GGAUGUUAUAUAUGAACCG |
| PTGER4 | CUGAGGACUUUGCGAAUAU |
| PTGER4 | GUGAAACACUGAACUUAUC |
| PTGER4 | CACUAACCAUGCCUAUUUC |
| PTGER4 | UAUAUAUCCUCCUGAGAAA |
