# Supplementary material for: Suppression of CCL2 angiocrine function by adrenomedullin promotes tumor growth
Source: J Exp Med. 2022 Nov 14;220(1):e20211628. doi: 10.1084/jem.20211628 (PMC9665902; doi:10.1084/jem.20211628)
Supplement: Table S2 — lists primer sequences used for qRT-PCR. [file JEM_20211628_TableS2.docx]

**Table S2.** Primer sequences used for qRT-PCR

| Gene | Forward (5′–3′) | Reverse (3′–5′) |
| --- | --- | --- |
| Human *ACP5* | CGGCCACGATCACAATCT | GCTTTGAGGGGTCCATGA |
| Human *ADAMTS9* | AACCCTGCGAGTATGTCTGG | TTCCACAGGTCACTGAGCAC |
| Human *ADM* | GCCTGCCCAGACCCTTAT | GTAGCGCTTGACTCGGATG |
| Human *ANKRD1* | GATCGAATTCCGTGATATGCT | AAACATCCAGGTTTCCTCCA |
| Human *ANTXR1* | TGTGTCCAGCGCCTATCTTA | AAAAGAGAGGCCATCGTTCAT |
| Human *ANXA2* | GAAAGTACGGCAAGTCCCTGT | CACCATTTCTGGACGCTCA |
| Human *ATP6AP2* | TATTGGGGAGGTGGGTTCCT | TCATAATGACAGCTGACAAGGG |
| Human *AXL* | AACCAGGACGACTCCATCC | AGCTCTGACCTCGTGCAGAT |
| Human *BGN* | CTCCCAGACCTCAAGCTCCT | TGGGACAGAAGTCGTTGACA |
| Human *BMP4* | TCCACAGCACTGGTCTTGAG | GGGATGTTCTCCAGATGTTCTT |
| Human *CAV2* | CCCTCAGCTGTCTGCACAT | TCACACTCTTCCATATTGTCTGC |
| Human *CCL2* | AGTCTCTGCCGCCCTTCT | GTGACTGGGGCATTGATTG |
| Human *COL4A1* | GACCCCCGGGAGAAATAG | CACTCCTGCAACACCATCTC |
| Human *COL4A2* | CCAGGACAGAAAGGAGACCA | GGTGTGATGCCTGGGAAC |
| Human *COL8A1* | TGATTCAGCAGTCCTGTCAAG | GGGAAATGGTAAGCAGCACT |
| Human *CRIM1* | CTACGTGCCCGAAGGAGA | CAGCCAGCGGGATTATTAAA |
| Human *CTGF* | CTCCTGCAGGCTAGAGAAGC | GATGCACTTTTTGCCCTTCTT |
| Human *CYR61* | AAGAAACCCGGATTTGTGAG | GCTGCATTTCTTGCCCTTT |
| Human *DKK1* | CAGGCGTGCAAATCTGTCT | AATGATTTTGATCAGAAGACACACATA |
| Human *DKK3* | CACATCTGTGGGAGACGAAG | CCCACAGTCCTCGTCGAT |
| Human *EDIL3* | ACCCAGAGGCTCAGAACAAC | TCGAAGACATTGCACTTTGC |
| Human *EDN1* | GCTCGTCCCTGATGGATAAA | CCATACGGAACAACGTGCT |
| Human *ESM1* | CATGGATGGCATGAAGTGTG | GGTGCCGTAGGGACAGTCT |
| Human *ESM1* | CAGTCTCAGGCATGGATGG | CTCACAATATTGCCATCTCCAG |
| Human *FLRT2* | GAGCGGGATTACCACCAG | TGGTTAAGCTCAATCTGCAATC |
| Human *GAPDH* | AGCCACATCGCTCAGACAC | GCCCAATACGACCAAATCC |
| Human *GNAS* | GCAGAAGGACAAGCAGGTCT | GCTTTTGCCAGACTCTCCAG |
| Human *ITGAV* | CATGTCCTCCTTATACAATTTTACTGG | GCAGCTACAGAAAATCCGAAA |
| Human *LDB2* | GCCTGAAGACCTGCTTGTTT | GTTGTTGGTTGCCTTGTGG |
| Human *LYVE1* | GAAGCAGCTGGGTTTGGAG | CGTAGCAAACAGCCAGCAC |
| Human *LPP* | TTCACCTGCGTGATGTGC | GCGGGGCAAATTTCTTGT |
| Human *MARCKSL1* | TGCATAAGGCAGTTGTTGGT | GGGATTTGGGGAGTAGGGTA |
| Human *MMP1* | GCTAACCTTTGATGCTATAACTACGA | TTTGTGCGCATGTAGAATCTG |
| Human *MMP2* | ATAACCTGGATGCCGTCGT | AGGCACCCTTGAAGAAGTAGC |
| Human *MYL12B* | CCTGTGCCCAACACTATCCT | TTTGCTCGACATGGTGGTT |
| Human *NMT2* | GAACTGGACGACCAGGACAC | TTTTGGCTCCCAAATACCC |
| Human *OSTM1* | GCAGATAGAATGCAAATAGTTGTGA | GCTGTTTGATAATTCTTCACTGTTGT |
| Human *PAWR* | GCAGATCGAGAAGAGGAAGC | TCATCTTCGTACTCATCTAAGCACTC |
| Human *PDGFB* | CTGGCATGCAAGTGTGAGAC | CGAATGGTCACCCGAGTTT |
| Human *PODXL* | GCGCTGCTGCTACTGTTGT | CCGTAGTAGTCTGGGTTGCAT |
| Human *PLXNA2* | GAGATCTACCTGACCCGGCTA | AAGTCGTCCACAAACTTCTGC |
| Human *RALGAPA2* | AAAACACGTAAAGGTCTCATTTCA | GCTCTTCGAGGTCAGCACTC |
| Human *SDCBP* | TCCAGCAATTTTGTCAGAAGC | GCTCTGGATACAGTCTGGGATAG |
| Human *SERPINE1* | AAGGCACCTCTGAGAACTTCA | CCCAGGACTAGGCAGGTG |
| Human *SETD7* | ATGGATAGCGACGACGAGA | TAATCCGTCATCGTCCAGGT |
| Human *SLC40A1* | GCTCTAGCTGTGAAAGCTGGTC | AGTTCCCTCCAGGGGTTTT |
| Human *SAMD4A* | TTCCTATTTACAGCTCATAGACAAGTG | GAGGGAAAGACCGAAAGAGC |
| Human *SYT11* | ACCAATATCCGACCTAGCTTTG | GACACACACCACCAGCACA |
| Human *TGFB2* | CCAAAGGGTACAATGCCAAC | CAGATGCTTCTGGATTTATGGTATT |
| Human *THBS1* | GCCACAGTTCCTGATGGAG | CCATGGAGACCAGCCATC |
| Human *TPM1* | GAGAACGCCTTGGATCGAG | CACCCGCAGCAACTTCTC |
| Human *TPM1* | CTGGACAAATACTCTGAGGCTCT | CTACGTCGGCTTCAGCATC |
| Human *TXNRD2* | CAGCGGGACTATGATCTCCT | AGGTTCCACGTAGTCCACCA |
| Human *UHMK1* | CTTCAAAGAAGGCAATCAGGA | GCCAAGCAATTTTGCAATTC |
| Murine *Adm* | TTCGCAGTTCCGAAAGAAGT | GGTAGCTGCTGGATGCTTGT |
| Murine *Ccl2* | CATCCACGTGTTGGCTCA | GATCATCTTGCTGGTGAATGAGT |
| Murine *Cd31* | CGGTGTTCAGCGAGATCC | ACTCGACAGGATGGAAATCAC |
| Murine *Gapdh* | CCATTTTGTCTACGGGACGA | GGGTTCCTATAAATACGGACTGC |
